# Structure and dimerization properties of the plant-specific copper chaperone CCH

**DOI:** 10.1038/s41598-024-69532-y

**Published:** 2024-08-17

**Authors:** Dominik Dluhosch, Lisa Sophie Kersten, Stephan Schott-Verdugo, Claudia Hoppen, Melanie Schwarten, Dieter Willbold, Holger Gohlke, Georg Groth

**Affiliations:** 1 Institute of Biochemical Plant Physiology, Heinrich-Heine-Universität Düsseldorf, 40225 Düsseldorf, Germany; 2 Institute for Pharmaceutical and Medicinal Chemistry, Heinrich-Heine-Universität Düsseldorf, 40225 Düsseldorf, Germany; 3 Institute of Bio- and Geosciences: Bioinformatics (IBG-4), Forschungszentrum Jülich, 52425 Jülich, Germany; 4 Institute of Biological Information Processing: Structural Biochemistry (IBI-7), Forschungszentrum Jülich, 52425 Jülich, Germany; 5 Institut Für Physikalische Biologie, Heinrich-Heine-Universität Düsseldorf, 40225 Düsseldorf, Germany

**Keywords:** Metallochaperone, Plant copper chaperone, ATX1 family, Dimerization, Protein–protein interaction, Biochemistry, Biophysics, Plant sciences, Structural biology

## Abstract

Copper chaperones of the ATX1 family are found in a wide range of organisms where these essential soluble carriers strictly control the transport of monovalent copper across the cytoplasm to various targets in diverse cellular compartments thereby preventing detrimental radical formation catalyzed by the free metal ion. Notably, the ATX1 family in plants contains two distinct forms of the cellular copper carrier. In addition to ATX1 having orthologs in other species, they also contain the copper chaperone CCH. The latter features an extra C-terminal extension whose function is still unknown. The secondary structure of this extension was predicted to be disordered in previous studies, although this has not been experimentally confirmed. Solution NMR studies on purified CCH presented in this study disclose that this region is intrinsically disordered regardless of the chaperone’s copper loading state. Further biophysical analyses of the purified metallochaperone provide evidence that the C-terminal extension stabilizes chaperone dimerization in the copper-free and copper-bound states. A variant of CCH lacking the C-terminal extension, termed CCHΔ, shows weaker dimerization but similar copper binding. Computational studies further corroborate the stabilizing role of the C-terminal extension in chaperone dimerization and identify key residues that are vital to maintaining dimer stability.

## Introduction

The redox-active transition metal copper is essential for a variety of cellular functions. Since copper ions can exist and switch between different redox states, the metal is typically found in enzymes such as amine oxidases^1^, proteins of the respiratory electron transport chain^2^, and in certain receptors responsible for the detection of gaseous molecules^2–4^. In plants, copper ions take on additional tasks as cofactors of photosynthetic protein complexes or sensors of the plant hormone ethylene^4^. More importantly, copper here is also an essential cofactor of superoxide dismutase (SOD) which is responsible for the detoxification of reactive oxygen species (ROS)^5,6^, e.g. in consequence of excess electron transfer in photosynthesis. Common to all organisms, copper ions are often found as electron acceptors and donators in proteins^7^. However, the redox-active properties of copper ions also bear the risk of ROS formation by Haber–Weiss/Fenton reactions^8^ which in turn can lead to oxidative damage of cell components^9^.

To avoid these detrimental side reactions, intracellular copper levels and copper transport have to be tightly regulated. Hence, many organisms have evolved copper chaperones to bind and shield copper ions while they are transported to their final target site^10,11^. Soluble copper chaperones of the Antioxidant-1 (ATX1) family share an overall similar structure consisting of a ferredoxin-like fold (βαββαβ) and a conserved MxCxxC motif that acts as a high-affinity tight binding site for monovalent copper ions^12^. These affinities are in the attomolar range, which has been confirmed in recent studies on human antioxidant 1 copper chaperone (ATOX1) and yeast ATX1 copper chaperones^13^. As a consequence, virtually no free copper ions are present within a cell, further emphasizing the highly effective regulation of free copper in cells^14^. Soluble copper chaperones are presumed to predominantly accept and release their metal cargo to specific targets such as membrane-bound heavy metal-transporting ATPases (HMAs). The interaction between the soluble chaperones and their dedicated targets is proposed to be accomplished by opposing surface charge distributions that are conserved between interaction partners^10,15^. In plants, direct interactions of ATX1-like chaperones with membrane transporter HMA7 (also known as RAN1) and HMA5 have been previously shown.

Recent studies in our lab revealed that ATX1-like chaperones also directly interact with the ethylene receptor family, a major group of copper proteins in plants^16–18^. A unique feature of vascular plants is that they possess two isoforms of soluble copper chaperones, ATX1 and CCH^18,19^. The two isoforms differ by the C-terminal extension in CCH of about 40 amino acid residues, but otherwise both share the same homology to other ATX1-like proteins in the N-terminal domain^19^. Complementary roles for both chaperones have been suggested based on transcription studies at different copper stresses in *A. thaliana*. Both chaperones were shown to be inversely regulated in plants grown in a medium containing either 50 µM CuSO_4_ (excess copper condition) and in plants grown in a medium containing 50 µM of the Cu(I)-specific chelator bathocuproine disulfonate (BCS) (copper deficiency conditions), respectively^20^. Further analyses disclosed that an intact MxCxxC metal binding motif and tight regulation of expression are required for maintaining plant copper homeostasis by ATX1, while CCH does not seem to act in a similar manner^21^ and is thus assumed to play a critical role in intercellular rather than in intracellular copper transport^22^. Structural analyses by far-ultraviolet (UV) and circular dichroism (CD) spectroscopy on full-length CCH and CCH subdomains propose that the chaperone indeed consists of two structurally independent domains with the isolated C-terminus being prone to temperature-induced changes in secondary structure^23^. Further structural analysis showed that the isolated C-terminus is able to form amyloid-like fibrils via ionic self-complementation^24^. Sequence-based secondary structure predictions propose that the C-terminus adopts an α-helical structure, whereas experimental studies by CD spectroscopy on a peptide derived from the C-terminal sequence point to a β-sheet conformation with amphipathic properties able to form macromolecular complexes by ionic complementation^23,24^. Up to now, structural information of the CCH C-terminal domain were exclusively obtained with the isolated domain or a short peptide corresponding to this domain, but not on full-length CCH. This raises the question, if the C-terminal domain follows the same structural, biochemical, and biophysical characteristics in the intact full-length protein.

To address this question, we studied full-length CCH and CCHΔ—a truncation mutant lacking the C-terminal extension. To this end, copper chaperones CCH and CCHΔ from *Arabidopsis* were expressed in *Escherichia coli*, purified to homogeneity and used for in vitro structural and interaction studies. In order to gain detailed structural information, solution nuclear magnetic resonance (NMR) studies on [*U*-^13^C, ^15^N]-labeled full-length CCH were performed. Purified proteins were further analyzed by microscale thermophoresis (MST) and absorption spectroscopy for their ability to form complexes/dimerize in a copper-dependent manner and for their copper binding properties. Finally, molecular simulations and free energy computations were performed using structures based on predictions by the deep learning-based algorithm *AlphaFold*^25,26^ to provide atomistic-level insights for the experimental findings.

## Results and discussion

### Solution NMR spectroscopy of CCH

**Figure 1:**
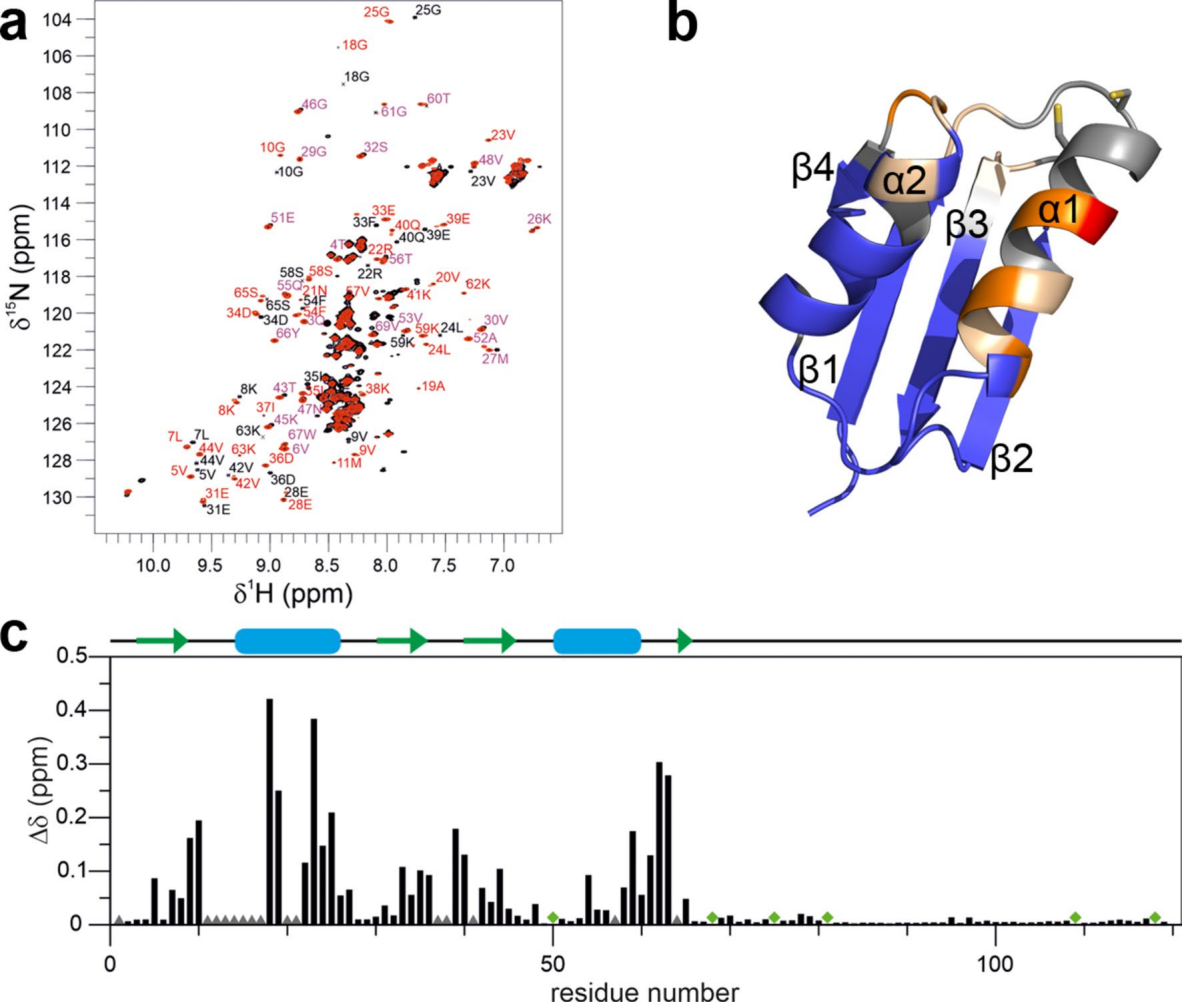
^1^H-^15^N HSQC spectra of CCH. (**a**) NMR spectra of CCH were recorded in the presence (black) and absence of Cu(I) (red). Assignments of the N-terminal domain are indicated in black for signals in the presence of Cu(I), red for signals in the absence of Cu(I) and purple for overlapping signals in both states. (**b**) Cartoon representation of the CCHΔ model based on the *AlphaFold* CCH model (identifier: **O82089**) showing regions affected by Cu(I) binding in orange shades. The cysteine sidechains are shown as sticks. (**c**) Weighted ^1^H and ^15^N chemical shift differences, Δδ, between resonances from the Cu^+^-CCH and apo-CCH are shown. Prolines are marked with a green diamond whereas residues that were broadened beyond detection in at least one state are indicated by a grey triangle. A schematic representation of the secondary structure based on the *AlphaFold* model is presented at the top.

**Figure 2:**
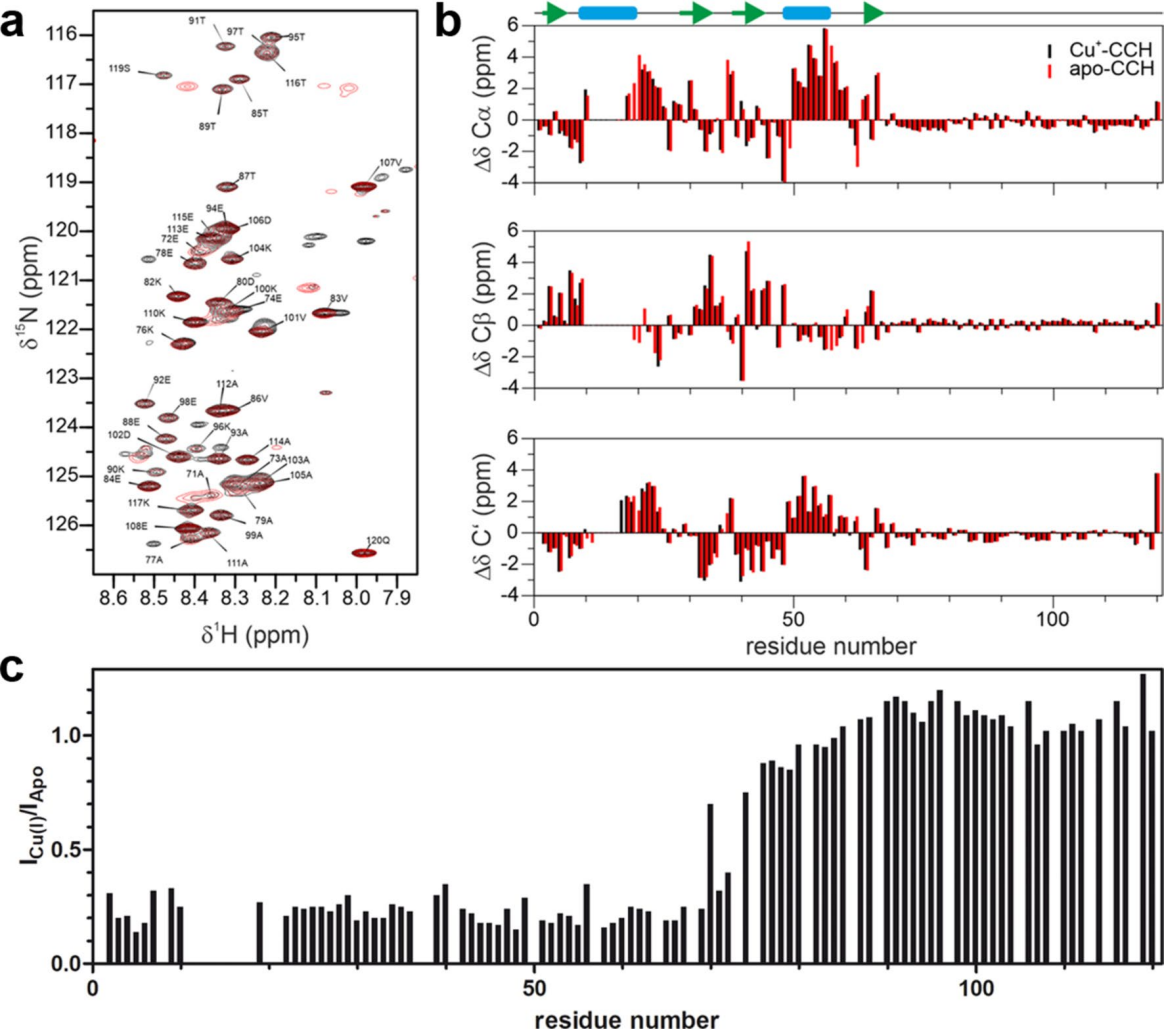
The C-terminal region of CCH is intrinsically disordered. (**a**) Overlayed ^1^H-^15^N HSQC spectra of Cu^+^-CCH (black) and apo-CCH (red) show that signals of residues belonging to the C-terminal region show a very low dispersion in the ^1^H dimension. (**b**) C_α_, C_β_ and Carbonyl secondary chemical shifts of CCH. A schematic representation of the secondary structure based on the *AlphaFold* model is presented at the top. (**c**) The signal intensity ratios in the N-terminal domain of CCH decrease in the Cu(I)-bound state compared to the copper-free state while the signal intensities in the C-terminal domain stay nearly unchanged indicating no direct involvement of the C-terminal domain in Cu(I)-binding.

To gain structural information on the C-terminal extension, solution NMR studies on full-length CCH were performed. From these studies backbone assignments for Cu(I)-CCH and apo-CCH were obtained. Unfortunately, signals of the copper binding motif MxCxxC were broadened beyond detection in these studies, most likely due to structural dynamics on an intermediate timescale and ligand exchange in this region or partial oxidation of the metal ion and formation of Cu(II), which would be paramagnetic and therefore broaden NMR of residues nearby. The biggest chemical shift changes upon chelating the Cu(I) ion were found in helix α2 following the MxCxxC motif (Fig. 1). Still, signals corresponding to the C-terminus were fully assigned and showed no significant difference between the Cu(I)-loaded and apo-state (Fig. 2a). The N-terminal region of CCH is structured and exhibits a secondary structure pattern similar to available structures of homologs (e.g., yeast ATX1), although helix α1 seems to be longer in CCH as seen by secondary chemical shifts analysis (Fig. 2b). In contrast, the signals for the C-terminal extension point for an overall unstructured domain, regardless of the addition of copper. Further, we compared the signal intensities in the ^1^H-^15^N HSQC spectra of the apo and the Cu(I)-bound state. For the C-terminal region, signal intensities stay nearly unchanged for the Cu(I)-bound and the apo form (I_Cu(I)_/I_apo_ ratios close to 1), indicating no direct involvement of the intrinsically disordered C-terminal domain in Cu(I) binding. However, we observe a significant decrease in intensities for the N-terminal domain upon Cu(I) binding (I_Cu(I)_/I_apo_ <  < 1, Fig. 2c), indicating an involvement of the globular CCH N-terminal domain in Cu(I) binding. In contrast to previous computational modeling and indirect structural approaches by far-UV and CD spectroscopy on the isolated C-terminus, our solution NMR spectroscopic analyses for the first time provide direct structural restraints of the CCH metallochaperone. The obtained NMR data clearly demonstrate that the C-terminal extension of CCH is intrinsically unstructured, in line with *AlphaFold*^25,26^ 3D structure predictions (see below) and sequence-based disorder analyses as can be seen by the reduced dispersion of the chemical shifts in that region (Fig. 2).

### Dimerization of CCH and CCHΔ studied by microscale thermophoresis

**Figure 3:**
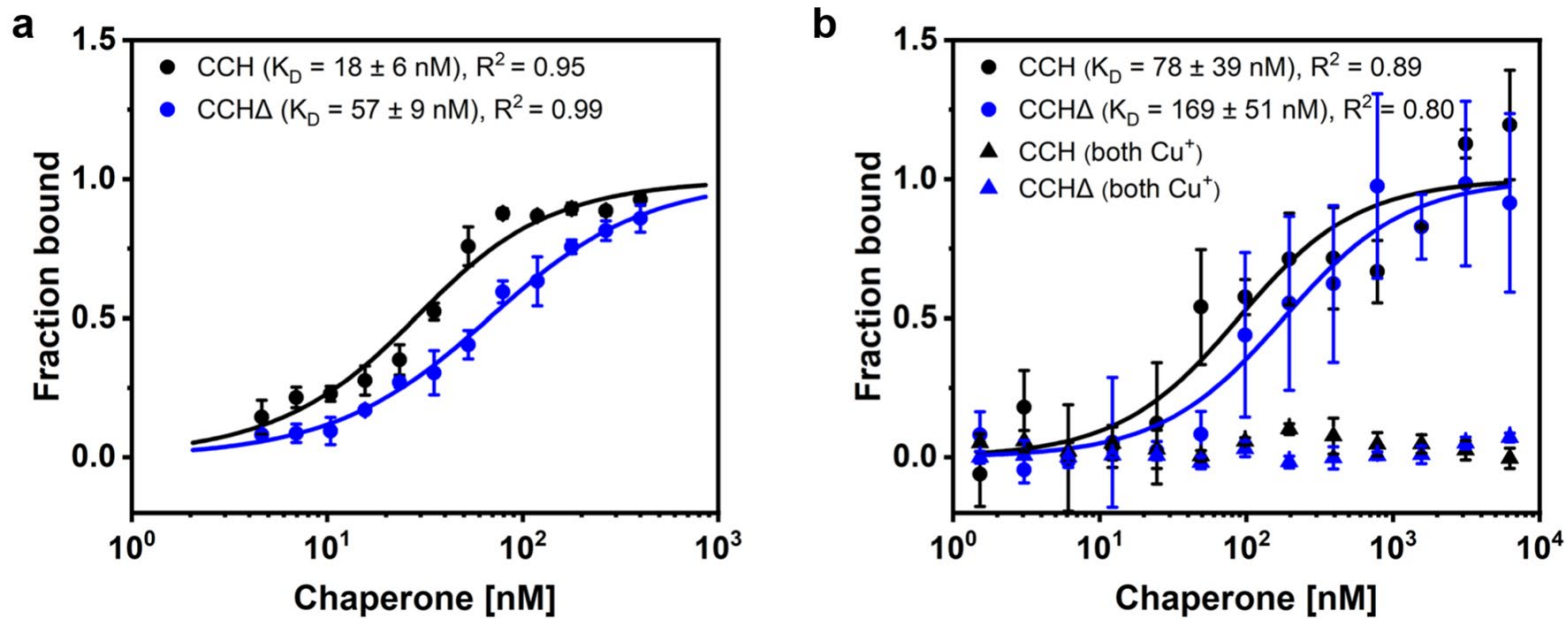
Homodimerization of CCH and CCHΔ under different copper loading states by microscale thermophoresis (MST). (**a**) Dimerization studies with both interacting proteins in their apo-state revealed an apparent dissociation constant (*K*_D_) of 18 ± 6 nM for CCH and 57 ± 9 nM for CCHΔ. (**b**) No dimerization was observed when both proteins were in their copper loaded state. Homodimerization was restored when only one monomer was in the copper-loaded state resulting in an apparent *K*_D_ of 78 ± 39 nM for CCH and an apparent *K*_D_ of 169 ± 51 nM for CCHΔ. Data indicate that the C-terminal extension of CCH is not required for dimerization, but suggest a stabilizing effect on dimerization as higher dimerization affinities were observed for CCH compared to CCHΔ in the apo- and copper loaded state.

Protein interaction studies based on microscale thermophoresis were used to analyze chaperone dimerization. When labeled CCH was titrated with unlabeled CCH, high-affinity binding was observed for the homomeric interaction of both proteins in the apo state with an apparent dissociation constant of *K*_D_ = 18 ± 6 nM (Fig. 3a). Interestingly, a similar behavior was observed when labeled CCHΔ was titrated with unlabeled CCHΔ (both chaperones in their apo state). However, a lower affinity constant with an apparent *K*_D_ value of 57 ± 9 nM was obtained for this interaction (Fig. 3a). The tighter binding observed for CCH suggests that the C-terminus contributes to dimer formation either directly by interaction with the N-terminus or C-terminus in the interacting monomer or indirectly by stabilization of the chaperone-chaperone interaction site. Direct interaction of both C-termini is supported by previous studies showing fibril formation of the isolated C-terminus^24^.

Next, we tested the homomeric interaction of both chaperone variants in their copper-loaded state. Here, no binding was observed, neither for CCH nor for CCHΔ (Fig. 3b). Based on these results we conclude that the copper binding site must play an important role in CCH dimer formation. This hypothesis is further supported by previous structural and biochemical work on copper chaperones of the ATX1 family (e.g., human ATOX1) where dimer formation is bridged by a single copper ion connecting the metal binding site in both monomers^27^. Naturally, dimerization is hampered—as observed in our studies—when both monomers are preloaded and saturated with copper as this would result in direct contact of two copper ions at the dimerization interface and unfavorable coulomb repulsion at this site. To test this idea, protein interaction studies were performed with one monomer in the copper-loaded and the other in the copper-free state. According to our assumption, chaperone-chaperone interaction was restored although with lower affinity. For CCH, the apparent *K*_D_ value was shifted to 78 ± 39 nM, for CCHΔ the apparent *K*_D_ became 169 ± 51 nM (Fig. 3b). Hence, in both cases, the affinity is lowered by a factor of 3–4 compared to the situation when both variants are in their apo-state. We propose that the observed reduced dimer affinity upon copper binding facilitates interaction of the copper chaperone and subsequent metal transfer to their membrane-bound target.

Copper-dependent interaction has been demonstrated for different types of copper chaperones before, such as the putative nuclear copper chaperone CCP^28^ or the cytosolic human ATX1 homolog HAH1/ATOX1^29^. In agreement with the MST binding studies presented in this work, previous studies from our lab^30^ by microfluidic diffusional sizing (MDS) point to copper-dependent dimerization of CCH. In contrast, these studies did not indicate metal-dependent interaction of CCHΔ as shown here, which may be related to the use of dithiothreitol (DTT) in these studies while the MST experiments were run in the absence of DTT. Such DTT dependence of metallochaperone interaction was previously observed^31^ for copper chaperone CopZ from *Bacillus subtilis*, which only dimerizes in the presence of copper when DTT is absent. This is probably because the redox agent can bind to protein-bound copper on its own due to its high affinity for Cu(I)-ions with the apparent *K*_D_-values of DTT for Cu(I) being in the fM-aM range^13^, thereby blocking potential interactions with another chaperone^32,33^. Studies on apo-ATOX1 revealed that the copper chaperone in the absence of DTT forms dimers when exposed to air^34^, but stays in the monomeric state when fully reduced and kept under anaerobic conditions. These studies are in line with the dimerization of CCH and CCHΔ in their copper-free state observed by MST. Similar to our results, previous studies on copper chaperone HAH1^35^ have observed dimerization in a copper-dependent manner and in the copper-free state with a reported apparent *K*_D_ for the copper-dependent homodimerization of HAH1 of 6 µM^35^ determined by surface plasmon resonance (SPR), which requires immobilization of one of the interacting proteins. In contrast, our interaction studies were performed in solution which could explain the higher dimerization affinities of CCH and CCHΔ due to the lack of steric hindrance introduced by the immobilizing surface required in techniques such as SPR. Still, it cannot be ruled out that dimerization of the chaperones in their apo-state then is owed to oxidation of the copper binding motif or carry-over of the metal ion from the bacterial expression host^36,37^. The high copper binding affinities in the attomolar concentration range reported for human ATOX1 and yeast ATX1^13^, which probably also apply for the *Arabidopsis* homolog CCH, ensure efficient loading even at trace amounts and transfer of the apo- to the copper-loaded state. To the best of our knowledge the cellular concentration of CCH in *Arabidopsis* has not been determined experimentally yet. Nonetheless, estimates of the cellular CCH concentration based on assumptions and data available in the literature^14,18,19,38^ lead us to the conclusion that our data are of physiological relevance due to estimated cellular concentrations of CCH varying between ~ 10 nM to 1000 nM. In this concentration range, monomeric and dimeric forms of the copper chaperones are present, underlining the physiological relevance of our data. The assumptions for the determination of cellular CCH concentrations are explained in more detail in the following chapter and in the "Methods" section.

### Estimating the ratio of monomeric and dimeric CCH- and CCH∆-dimer in the cell

**Figure 4:**
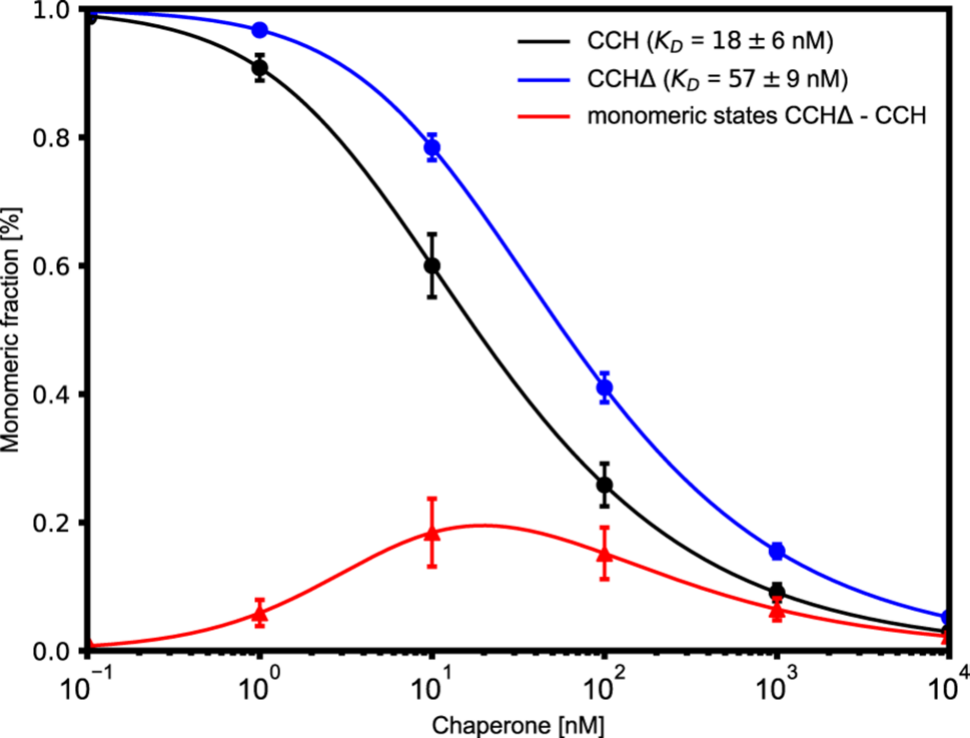
Chaperone concentration and the corresponding fraction of monomeric states of CCH- and CCH∆-dimers. Under the anticipated physiological conditions of 10 to 1000 nM both the monomeric and dimeric states are present for CCH and CCH∆. However, particulary in that range, the amount of CCH∆ monomers is up to 20% higher than the amount if CCH monomers, which shows that the different *K*_D_ values and thus, the C-terminal end have an impact on the monomeric and dimeric states of the chaperones CCH.

To estimate if the dimerization of the CCH-dimer is dependent on the unstructured C-terminal end, *K*_D_ values of CCH (*K*_D_ = 18 nM) and CCH∆ (*K*_D_ = 57 nM) were determined experimentally. To our knowledge, no physiological concentration of CCH has been measured in *Arabidopsis thaliana* so far. Therefore, we assumed that the physiological concentration of CCH molecules in the cell is one CCH molecule per 10 to 1000 SOD1 molecules, i.e., the CCH concentration is ~ 10 to ~ 1000 nM. This assumption is based on that for SOD1, the intracellular concentration has been measured in yeast^14,18,19,38^. In *Arabidopsis thaliana*, SOD1 is present too, and suggested to transport copper to the chloroplast. The chloroplast acts as a Cu storage such that we presume that SOD1 is present in considerably higher concentrations than CCH to fulfill the task. Under such conditions, and considering the equilibrium of dimer-to-monomer transition, between 60 and 9% and 78 and 16% of the CCH- and CCH∆ molecules are predicted to be in a monomeric state. The results show that under these conditions, both the monomeric and dimeric states are present for CCH and CCH∆. However, the difference corresponds to 18 to 7% more monomeric states for CCH∆ than for full-length CCH. Figure 4 illustrates the chaperone concentration from 10^–1^ to 10^5^ nM and the corresponding proportion of monomers for their *K*_D_ values. Particularly in the range from 10 to 1000 nM, the amount of CCH∆ monomers is higher than the amount of CCH monomers, which shows that the different *K*_D_ values and, thus, the C-terminal end have an impact on the monomeric and dimeric states of CCH(∆) under the anticipated physiological conditions. Our results indicate that CCH due to the C-terminal end behaves differently than CCH∆ in the assumed range of physiological concentrations and, thus, presumably also than its homolog ATX1. This might explain, why CCH and ATX1 are hypothesized to function differently and, therefore, also perform different tasks in the cell^18^.

### Influence of the C-terminal extension on protein stability

**Figure 5:**
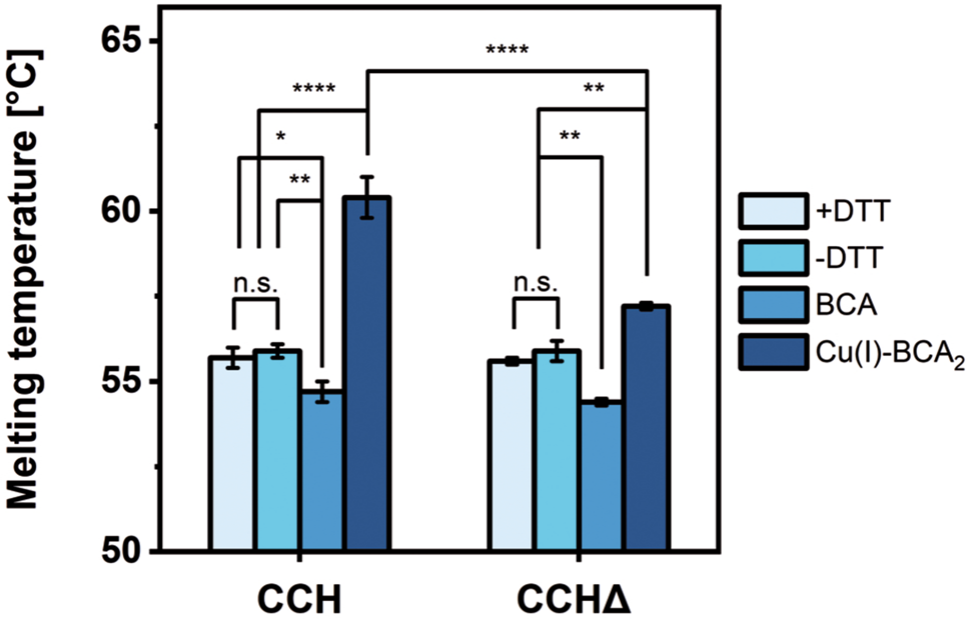
Graphical representation of melting temperatures of CCH and CCHΔ under different conditions obtained by nanoDSF. Significant increases in the melting temperatures of CCH and CCHΔ were observed in the presence of copper. The melting temperature CCH increases significantly more than that of CCHΔ in the presence of copper demonstrating a stabilizing effect of the C-terminal extension. No significant differences were found for chaperones treated with DTT (+ DTT) compared to the non-treated (-DTT) samples. Error bars represent the standard deviation (s.d.) of the mean of technical triplicates (n = 3). Asterisks indicate significance levels determined by two-way analysis of variance (ANOVA) and pairwise multiple comparisons using the post-hoc Bonferroni test (no significance (n.s.), *p* ≤ 0.05 (*), *p* ≤ 0.01 (**), *p* ≤ 0.0001 (****)).

To further support the hypothesis of a stabilizing effect of the C-terminal extension, nano differential scanning fluorimetry (nanoDSF) was used to study protein stability in the presence and absence of copper, free bicinchoninic acid (BCA), and the redox reagent DTT. Statistical analysis shows a significant increase in the melting temperature (*T*_m_) of CCH and CCHΔ by copper (Fig. 5, Table 1 and Supplementary Fig. S1 online), which increases from 55.9 °C (samples without DTT) to 60.4 °C (CCH) and 57.2 °C (CCHΔ). The increase in the melting temperature of CCH is significantly (*p* ≤ 0.0001) stronger than that of CCHΔ compared to the melting temperatures of the individual proteins in the absence or presence of DTT, clearly demonstrating a positive effect of the C-terminal extension on protein stability. To exclude stabilization of copper chaperones by the copper ligand BCA, protein stability was analyzed in the presence of BCA only. As can be seen in Fig. 5 and Supplementary Fig. S1 online as well as Table 1, free BCA decreases the stability of CCH and CCHΔ to a similar degree demonstrating that the stabilization observed for samples incubated with the Cu(I)-(BCA)_2_ complex is solely due to the copper ion. In contrast, the addition of DTT to the buffer did not alter the melting temperatures of CCH and CCHΔ significantly (*p* > 0.05). Moreover, both proteins show similar melting temperatures in their apo states. Overall, the data obtained by nanoDSF demonstrate a positive effect of the C-terminal extension on protein stability in the presence of copper.

**Table 1:** Effect of DTT, monovalent copper and free BCA on the melting temperatures of CCH and CCHΔ studied by nanoDSF.

Additive	Melting temperatures *T*_m_ [°C]		*n*
	CCH	CCHΔ	
+ DTT	55.7 ± 0.3	55.6 ± 0.1	3
- DTT	55.9 ± 0.2	55.9 ± 0.3	3
Cu(I)-(BCA)_2_	60.4 ± 0.6	57.2 ± 0.1	3
BCA	54.7 ± 0.3	54.4 ± 0.1	3

Monovalent copper was provided as the chromophoric Cu(I)-(BCA)_2_ complex. Melting temperatures (*T*_m_) are given as mean values with their corresponding standard deviation and n represents the number of technical replicates.

### Structural modeling of the CCH- and CCHΔ-dimers

To generate a structural model of the CCH- and CCH∆-dimers, *ColabFold* 1.5.2^39^ with default settings was used. To understand the evolutionary relationship of CCH and ATX1 of *A. thaliana*, both were sequentially aligned. The alignment reveals an *E*-value of 2 × 10^–43^, a query coverage of 59%, and a sequence identity of 78%, confirming homology, (Supplementary Fig. S2 online), as mentioned in ref.^21^. To identify homologous structures from other organisms, the BLAST suite^40,41^ was used for a search against the PDB database^42^. This identified homologous structures from *Homo sapiens, Oryza sativa, Saccharomyces cerevisiae,* and *Thermus thermophilus* with *E*-values, query coverages, and sequence identities ranging from 2 × 10^–9^ to 3 × 10^–6^, 54% to 46%, and 41% to 39%, respectively. These values, along with several conversed amino acids including the characteristic copper-binding motif (MxCxxC) (Supplementary Fig. S2 online), confirm that CCH is a homolog of ATX1 in humans and yeast, as mentioned in refs.^19,21,43,44^. For the sequence resulting in the lowest *E*-value (UniProt ID: **O00244**), the PDB structure shows a homodimeric ATX1 complexed with a cadmium ion (PDB ID: **1FE0**)^27^. However, another homodimer complexed with a copper ion was structurally resolved in the same publication (PDB ID: **1FEE**)^27^. Additionally, a heterodimeric ATX1 structure matching the UniProt sequence P38636 was found (PDB ID: **2GGP**)^45^. Therefore, the homodimeric ATOX1 complex from humans (PDB ID: 1FEE)^27^ and the heterodimeric Ccc2a-ATX1 complex from yeast (PDB ID: 2GGP)^45^ were used to validate the *ColabFold* models.

Based on the full-length sequence of CCH, *ColabFold* predicted a dimer in which the individual monomers feature the typical ATX1-like fold, but failed to predict dimer structures in which the Cu(I) coordinating residues Cys13 and Cys16 are facing towards each other as anticipated from homolog structures found in the Protein Data Bank (PDB)^42^ (*Saccharomyces cerevisiae*, Atx1-Cu(I)-Ccc2a complex, PDB ID: **2GGP**)^45^, (*Homo sapiens*, COPPER-HAH1, PDB ID: **1FEE**)^27^. The pLDDT (predicted local distance difference test; a per-residue confidence metric) indicates the high accuracy of the models, especially for the residues 1–66 (Supplementary Fig. S3 online). In this region the sequence coverage is high (Supplementary Fig. S3 online), indicating a good structure prediction of the monomers based on evolutionary information. However, the drop in the pLDDT and the sequence coverage for residues 67 and the following indicates that the structural information of the C-terminal part of CCH is not well defined.

To gain further insights into the structure of CCH, predictions were performed using *TopSuite*, a deep-learning meta-server for computational structural biology^46^. *TopDomain*^47^ performs a domain boundary prediction for full-length CCH. A boundary at position 72 with a peak score of 0.85 is predicted. This predicted boundary agrees with the drop of PAE and pLDDT at position 67 in the *ColabFold* models (Supplementary Fig. S3 online). The neural network-based tools for the prediction of disordered regions *TopProperty*^48^ and DISOPRED^49^ show that the C-terminal region of CCH has a high tendency to be disordered (Supplementary Fig. S3 online). Taking the above together, we thus assumed that *ColabFold* fails to predict the correct CCH dimer configuration due to the lack of order in the C-terminus structure. Furthermore, from the structure prediction methods, no information is available if the C-terminus is involved in the formation of the dimer interface, nor if the termini interact with each other or the monomers. However, by omitting residues 72 to 121 of the CCH sequence, models of the CCH∆-dimer that fulfill the structural arrangement known from the homologs could be generated with *ColabFold* 1.5.2^39^. The models show the typical ATX1-like folds for the monomers^50^. The PAE (predicted alignment error) and pLDDT indicate a high accuracy of the models, especially for residues 1–66 (Supplementary Fig. S4 online).

To validate the proposed models of the CCH∆-dimer, they were compared to the human homolog ATOX1 (PDB ID: **1FEE)**^27^. The rank 1-model shows the lowest C_α_ atom root-mean-square deviation (RMSD) to the crystal structure (0.5 Å). Additionally, the orientation of the side chains known to form the dimer interface is broadly consistent with the homolog, which is particularly true for the residues forming the Cu(I) binding site, Ser12, Cys13, and Cys16. Minor deviations exist for residues Arg22, Lys59, Lys62. No information is available about the orientation of Lys63 as there is a threonine residue in the homolog. For the homolog PDB ID: **1FEE**, it was suggested that the bound metal ion together with an intermonomer hydrogen bond network provide the key forces that hold the two monomers together^27^. Locations of water molecules in the dimer interface were calculated by 3D reference interaction site mode (3D-RISM) and agree with the water positions of the homolog PDB ID: **1FEE** (Supplementary Fig. S5 online).

Overall, the model of the CCH∆-dimer obtained by *ColabFold* and 3D-RISM agrees with the homologs at the level of global structure, indicated by the low C_α_ RMSD value, and a more detailed level, including the orientation of the residues forming the dimer interface, suggesting a reasonable structural prediction for the CCH∆-dimer. Finally, the disordered C-terminus from the full-length prediction (residues 72–121) of the CCH dimer was attached to the model of the CCH∆-dimer to obtain a model for the full-length CCH-dimer. Due to the disordered nature of the C-terminal end, the orientation of the C-terminus in the CCH-dimer model is weakly defined, particularly when compared to the well-defined ATX1-like fold and the dimer interface confirmed by experimental data from a human homolog. Therefore, MD simulations were performed to gain insights into the structural dynamics of the CCH dimer.

### Molecular dynamics simulations of the CCH- and CCHΔ-dimers

The dimer models were subjected to molecular dynamics (MD) simulations. For each model, fifty independent replicas were simulated for 200 ns each. The trajectories show a slight C_α_ atom RMSD drift of 1–3 Å concerning the corresponding starting structure for the respective monomers in the dimer, indicating that the ATX1-like folds remain invariant during the simulations. Two replicas of the CCH∆-dimer and three replicas of the CCH-dimer show a C_α_ atom RMSD of the dimer that is similar to that of the monomers (Supplementary Fig. S6 online). There, both monomers coordinate the Cu(I) ion via Cys13 and Cys16, and the hydrogen bond network^27^ is formed, corresponding to the “closed conformation” of the two conformational states of the copper metallochaperone ATOX1 based on electron paramagnetic resonance (EPR) data^51^ as well as EPR data and multiscale simulations^52^ (Supplementary Fig. S7 online). In eight to 24 replicas, the C_α_ atom RMSD is ~ 6 Å for the CCH∆-dimer and the CCH-dimer, respectively, indicating pronounced conformational changes, e.g., that the dimer is mainly held together by the copper-coordinating cysteines but that the hydrogen bond network is largely missing. Such structures correspond to the “open conformation” of the two conformational states of ATOX1^51,52^ (Supplementary Fig. S7 online). Finally, in 23–40 replicas, the C_α_ atom RMSD reaches at least 10 Å for the CCH-dimer and the CCH∆-dimer, respectively, due to dimers that dissociated and remain so or the monomers reassemble as a dimer, but in an alternate conformation, in which the respective copper-coordinating cysteines of each monomer do not point to each other, which prevents copper coordination of both monomers, similar to the “back-to-face” conformation^53^ (Supplementary Fig. S7 online). A trend is emerging that indicates that the CCH-dimer replicas provide more closed and open configurations, in total 27, compared to the CCHΔ-dimer with in total 10 replicas. As to the behavior of the C-termini of the CCH-dimer, no interaction of the C-termini with each other was observed. Either the C-termini are exposed to the solvent or located close to the monomer at the same side as the first β-strand of the ATX1-like fold with certain residues (80–95, and 103–121) showing short distances to the first α-helix (residues 18–25) of the other monomer (Supplementary Fig. S8 online).

To obtain more replicas of the CCH-dimers in the “closed conformation” for subsequently determining the dimer interface, the final structures of replicas 43 and 49 for the CCH- and CCH∆-dimer, respectively, were each minimized, thermalized, and subjected to production runs of another 10 independent MD simulations of 200 ns length each. In addition, replica 43 features small distances for both residues 80–95 and 103–121 of the C-termini to the dimer interface. This will allow the suggestion of key residues that contribute to the monomer association by a residue-wise decomposition of the binding effective energy (see below). In these extended trajectories, little to no conformational changes are observed (C_α_ atom RMSD 1–4 Å) (Supplementary Fig. S9 online). To generate conformational ensembles of the apo CCH- and CCHΔ-dimers, the same procedure was applied, but the Cu(I) ions were removed. As for the copper-loaded complexes, the trajectories show little to no conformational changes (C_α_ atom RMSD 1–4 Å) (Supplementary Fig. S9 online).

### Residue-wise decomposition of binding effective energies

**Figure 6:**
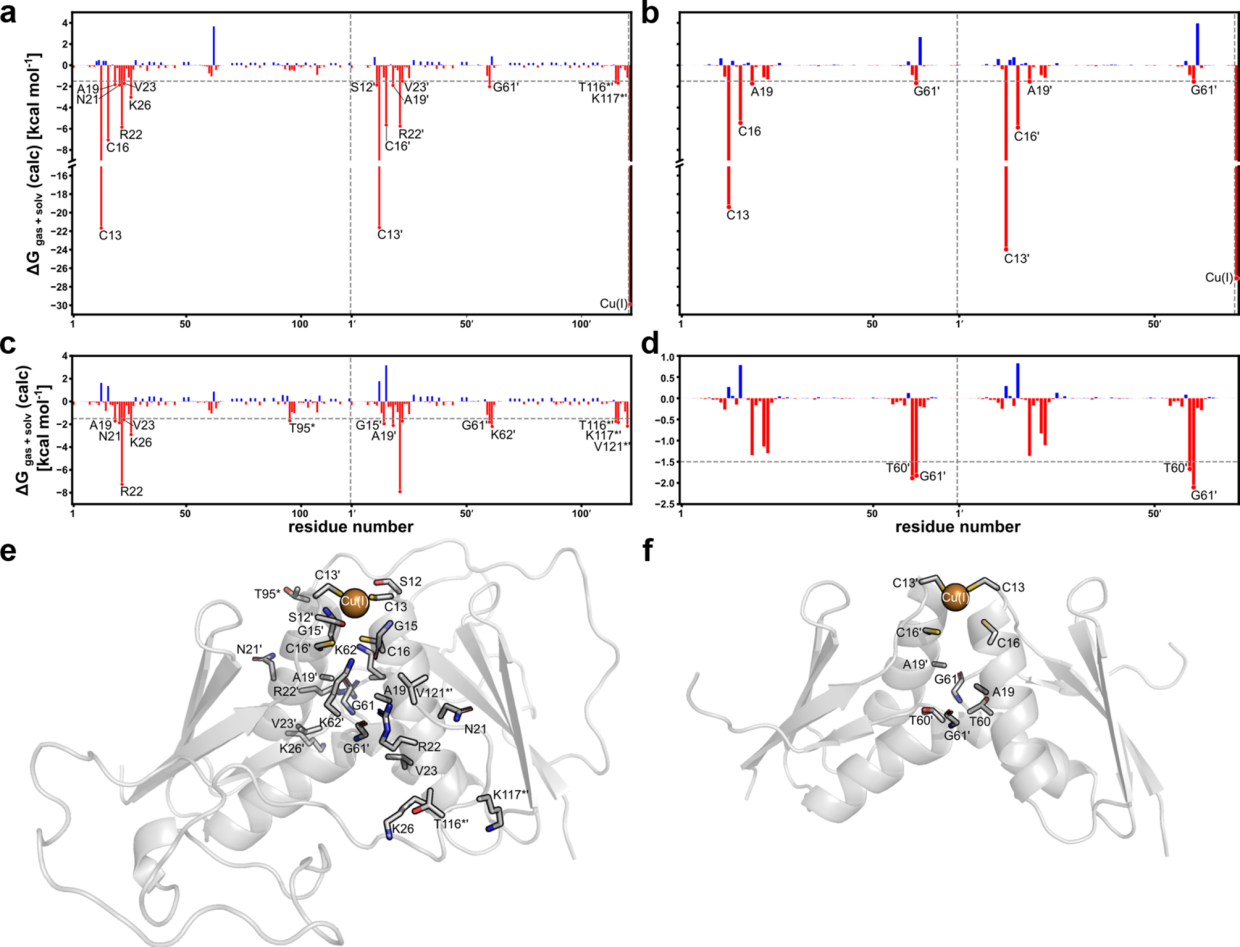
Identification of amino acids in CCH- and CCH∆-dimers that are crucial for dimer stability. (**a**) Per residue decomposition of the binding effective energy of the Cu(I)-loaded full-length CCH-dimer. Residues contributing to the dimerization with ∆*G* < -1.5 kcal mol^−1^ are considered hot spots and are indicated in the graphic by red dots. Residue numbering is according to the full-length CCH monomer (UniProt code: **O82089**). The dotted line separates the two protomer chains. The residues of the C-termini are marked by *. Residues discussed in the text are labeled. The relative standard error is < 2.5% of the corresponding hot spot mean. (**b**) Same as in (**a**) for the Cu(I)-loaded CCH∆-dimer. (**c**) Same as in (a) for the apo full-length CCH-dimer. (**d**) Same as in (**a**) for the apo CCH∆-dimer. (**e**) Hot spot residues localized on the full-length CCH-dimer. (**f**) Hot spot residues localized on the CCH∆-dimer. The energy decomposition revealed that favorable polar contributions to the solvation free energy occur around the Cu(I) ion and involve residues C13 and C16. R22 is also part of the hydrogen bond network and shows favorable contributions of electrostatic interactions since the guanidino group of the side chain forms a hydrogen bond with the carbonyl oxygen of G61 of the opposite monomer. The remaining polar (N21, K26) and nonpolar (A19, V23) amino acids are in the dimer interface but do not participate in the hydrogen bond network. They contribute to the stability of the dimer mainly due to van der Waals interactions and to a smaller extent through non-polar contributions to the solvation free energy, as do residues T95, T116, K117, and V121 located at the C-terminal end of CCH.

To identify amino acids in the CCH- and CCH∆-dimers that are crucial for dimer stability, molecular mechanics/generalized Born surface area (MM-GBSA)^54^ calculations combined with a decomposition of the effective energy of dimerization on a per-residue level were performed^55^. In the case of the copper-loaded CCH-dimer, 1000 snapshots from the ten extended replicas were used (Supplementary Fig. S9 online). Residues S12, C13, C16, A19, N21, R22, V23, K26, G61, and Cu(I) in the ATX1-like fold show ∆*G*_gas + solv_ < − 1.5 kcal mol^−1^ and contribute favorably to the binding effective energy of the complex. In the disordered C-termini, T116 and K117 of one monomer contribute favorably to the binding effective energy with ∆*G*_gas + solv_ < − 1.5 kcal mol^−1^ (Fig. 6a, e). In the case of the loaded CCH∆-dimer, 1000 snapshots from eight extended replicas were investigated (Supplementary Fig. S9 online). Less than half of the residues identified for the CCH-dimer (C13, C16, A19, G61, and Cu(I)) show ∆*G*_gas + solv_ < − 1.5 kcal mol^−1^ (Fig. 6b,f) now. In the case of the apo CCH-dimer, 1000 snapshots from six extended replicas were investigated. Residues G15, A19, N21, R22, V23, K26, G61, K62, T95, T116, K117, and V121 show ∆*G*_gas + solv_ <  - 1.5 kcal mol^−1^ (Fig. 6c,e). C13 and C16 are no longer binding “hot spots” due to the missing Cu(I) but additional residues in the C-terminus (T95, V121) now contribute favorably compared to the copper-loaded state. In the case of the apo CCH∆-dimer, 1000 snapshots from eight extended replicas were investigated. Residues T60 and G61 show ∆*G*_gas + solv_ < − 1.5 kcal mol^−1^ (Fig. 6d,f). As for the apo CCH-dimer, C13 and C16 are no longer binding “hot spots”. Due to the lack of the C-termini, there is no compensation by such residues possible**.**

### Copper binding of CCH and CCHΔ

**Figure 7:**
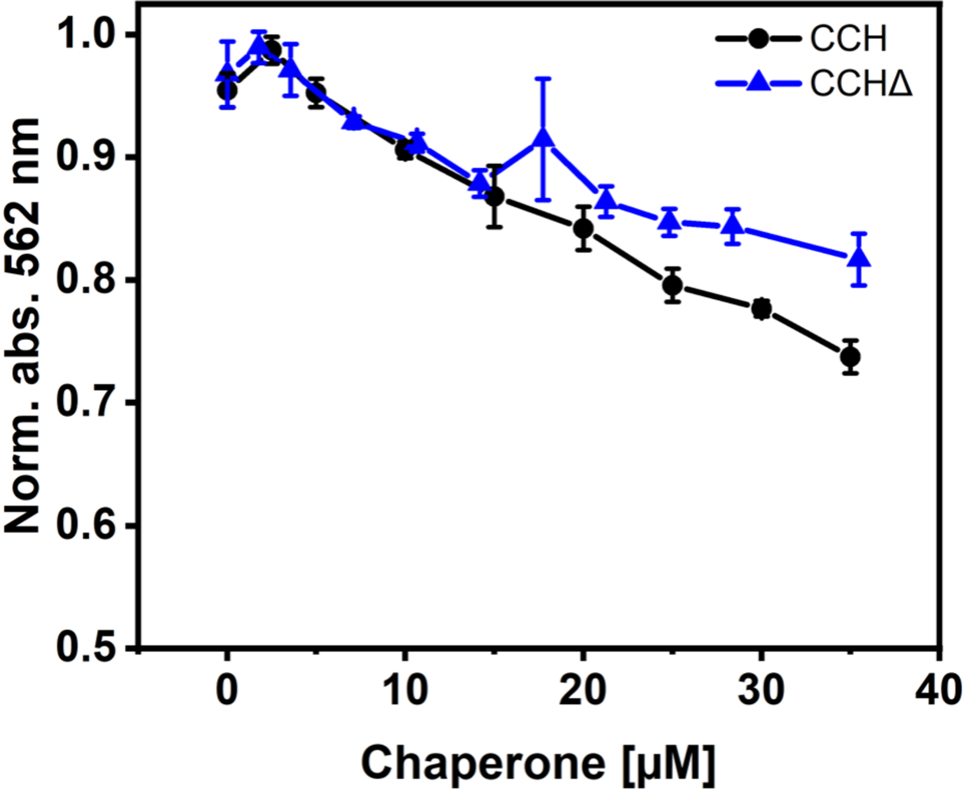
Titration of Cu(I)-(BCA)_2_ with CCH and CCHΔ. A fixed concentration of the chromophoric Cu(I)-(BCA)_2_-complex was titrated with different concentrations of copper chaperones CCH and CCHΔ in the same final volume and absorption at 562 nm was monitored. A decrease in absorption at increasing chaperone concentrations indicates removal of copper from the Cu(I)-(BCA)_2_-complex due to copper binding at CCH or CCHΔ, respectively.

To substantiate that the C-terminus has no direct impact on the metal binding site in the CCH N-terminal domain, copper binding of CCH and CCHΔ was studied with chromophoric copper-bicinchoninic acid complex Cu(I)-(BCA)_2_. The observed decrease in absorption at increasing concentrations of CCH and CCHΔ indicates that chaperones and high affinity ligand BCA compete for monovalent copper and that copper is transferred onto the soluble chaperones (Fig. 7). Similar changes were observed for CCH and CCHΔ especially at lower chaperone concentrations leading to the conclusion that the C-terminal extension has no or only a minor effect on copper binding efficiency. This conclusion is in line with the previously shown solution NMR experiments where no significant differences in the structure of the C-terminus were observed in the presence and absence of copper.

## Conclusion

Previous structural analysis of CCH and its C-terminal extension focused on isolated domains and short peptide sequences derived from the C-terminal extension. In contrast, the solution NMR-data in this study provide for the first time restraints and offer structural insights into the full-length protein. They demonstrate that the C-terminal extension is intrinsically disordered and further reveal that copper has no detectable influence on the structure of this extension. These new findings are in line with structure predictions by the deep learning algorithm *AlphaFold* and are further complemented by in vitro and in silico CCH dimerization studies in this work. From domain identification, structure, and disorder prediction, the C-terminal end is likely disordered. A per-residue decomposition of the binding effective energy revealed that residues in the dimer interface also known from homologous dimers predominantly determine the monomer association^27,45^. Residues involved in copper binding contribute in the copper-loaded state as do residues T116, K117, and V121 in the C-terminus. The latter are part of a group of residues that shows short distances in unbiased MD simulations of the CCH-dimer to the first α-helix of the other monomer. The influence of the C-terminal extension was also evident in the melting temperature analysis, which demonstrated that the melting temperature of CCH increased more than that of CCHΔ in the presence of copper. Our computational results are in line with experimental data that showed that CCH-dimer formation is more favorable in the presence of the C-termini (Fig. 3). Complementary biophysical studies on CCH interaction revealed tighter interaction of the monomers in the CCH dimer compared to the corresponding CCHΔ complex. Based on these findings, we propose an indirect effect of the C-terminal extension on the dimerization due to the stabilization of the dimer interface. The physiological function of chaperone dimerization might consist in an additional-shielding of the bound hazardous copper cargo providing another safety layer in the proposed long-range intercellular transport of monovalent copper by CCH^22^. In addition, dimerization may also protect the chaperone metal binding motif from unfavorable side reactions (e.g., oxidation). Our data suggests that CCH exhibits a distinct dimerization behavior compared to CCH∆, and probably also differs from its homolog ATX1, due to the C-terminal end. This aligns with the hypothesis that CCH and ATX1 have distinct functional roles, potentially performing different tasks within the cell^18^. The reduced dimerization affinity of CCH and CCHΔ in the copper-loaded state may ensure further interaction and copper transfer from the soluble chaperone to their membrane target. The precise nature and the molecular mechanism of the proposed indirect impact of the C-terminus on dimerization, however, could not be identified by the methods used in this study and require further analysis. Nevertheless, the experiments performed in this study demonstrate that the C-terminus of CCH is intrinsically disordered in the full-length protein and is not involved in Cu(I)-binding but affects the dimerization behavior of this copper chaperone and therefore potentially its cellular function.

## Methods

Recombinant proteins CCH and CCHΔ were expressed and purified as previously described^17^. The vectors used to do so consist of the coding sequence of CCH (TAIR: **AT3G56240.1**) for amino acids 1–120 for full-length CCH or for amino acids 1–70 for CCHΔ. Proteins are fused to an N-terminal 10xHis-tag, followed by a tobacco etch virus (TEV) protease cleavage site for His-tag removal after affinity purification. His-tag removal and loading of purified copper chaperones with monovalent copper was also performed as previously described in^17^. In short, copper chaperones were purified by immobilized metal ion affinity chromatography (IMAC). His-tag removal was achieved by the digestion of copper chaperones with TEV-protease prior to the final purification step by size exclusion chromatography (SEC). For NMR experiments a Superdex™ 200 (GE Healthcare, Illinois, USA) was used whereas otherwise a Superdex Increase 75™ 10/300 GL (Cytiva, Marlborough, USA) was used. For copper binding studies an additional purification step was included. His-tags were removed by overnight incubation with TEV-protease at 4 °C in a 1/70 molar ratio in buffer A (300 mM NaCl, 50 mM 2-[4-(2-Hydroxyethyl)piperazin-1-yl]ethane-1-sulfonic acid (HEPES), 10% (w/v) glycerol, 1 mM (DTT), pH 7.0–7.5). TEV-protease and uncleaved chaperones were removed by reverse IMAC on a ÄKTAPrime plus system (GE Healthcare) using a 5 ml HisTrap FF column (Cytiva, Marlborough, USA) equilibrated in buffer A. Flow-through fractions were collected and cleaved chaperones were eluted using 20 mM Imidazole in buffer A. Fractions were then pooled and concentrated to a final volume of 0.5 ml using Amicon Ultra-15 centrifugal filter units (Merck Millipore, Darmstadt, Germany) with a molecular weight cut-off of 3 kDa. Imidazole, protein aggregates and TEV-protease were removed by gel filtration using a Superdex™ 75 Increase size exclusion chromatography column (Cytiva, Marlborough, USA) equilibrated with degassed SEC buffer (300 mM NaCl, 50 mM HEPES, pH 7.5). Elution fractions were pooled, concentrated as described above and protein concentration was determined based on the absorption at 280 nm. Chaperones were adjusted to 20% (w/v) glycerol, shock frozen in liquid nitrogen and stored at -80 °C. All purification steps were carried out at 4 °C. For NMR-experiments, freshly prepared protein without the addition of glycerol was used. Buffers were prepared from ultrapure water with a conductivity of 0.055 µS/cm.

### Copper binding of chaperones

For analysis of copper binding abilities of CCH and CCHΔ, chaperones were incubated for 1 h at 4 °C with a 20-fold excess of DTT over protein in a reaction volume of 0.5 ml in degassed SEC buffer. DTT was then removed by buffer exchange into degassed SEC buffer using a PD MiniTrap desalting column (Cytiva, Marlborough, USA). Protein concentration was determined via absorption at 280 nm and a titration series of protein in SEC buffer was prepared ranging from 0 to 100 µM depending on the protein concentration in a volume of 50 µl. Afterwards, 50 µl of Cu-buffer (300 mM NaCl, 50 mM HEPES, 100 µM BCA, 40 µM CuCl, 2 mM Na-ascorbate, pH 7.5) were added to the protein dilution series and absorption at 562 nm was measured. All measurements were performed in triplicates at room temperature.

### Fluorescent labeling of purified chaperones

Interaction studies using microscale thermophoresis (MST) require labeling one of the interaction partners with a fluorescent dye. Therefore, CCH and CCHΔ, purified as previously described^17^, were labeled with the amine reactive dye Alexa Fluor™ 488 N-hydroxysuccinimide (NHS) ester by adding the threefold amount of dye in relation to protein in SEC buffer. Afterwards the reaction mixture was incubated for 1 h in the dark on a rotary shaker followed by the addition of a 1000-fold excess of a Tris/HCl pH 8.0 solution to quench the reaction. For experiments involving copper, TEV-protease (1/70 protease to chaperone ratio) and DTT at a final concentration of 1 mM were added followed by overnight incubation in the dark at 4 °C. Finally, Cu(I)-(BCA)_2_ was slowly added until the solution stayed colored and a sample volume of 0.5 ml was loaded on a Superdex™ 200 Increase 10/300 GL size exclusion chromatography column equilibrated in SEC buffer to remove excess dye, Cu(I)-(BCA)_2_ and TEV-protease^17^. Elution fractions were pooled, concentrated and protein concentration was determined as described above.

### Microscale thermophoresis

Interaction studies were performed on a Monolith NT.115 using premium capillaries (NanoTemper Technologies, Munich, Germany). Measurements were done in triplicates with MST power set to 70% and LED power set to 95%. Prior to use, all protein stock solutions were adjusted to 0.05% (w/v) Tween-20 and all dilutions were made with MST buffer (300 mM NaCl, 50 mM HEPES, 0.05% (w/v) Tween-20, pH 7.5). In all measurements, labeled chaperones were diluted to a final concentration of 20 nM. For studying unloaded CCH-CCH and CCHΔ-CCHΔ homomeric protein interactions, a 2:1 dilution series of unlabeled protein ranging from 4.6 to 400 nM was prepared. For studying the protein interaction of chaperones preloaded with copper, dilutions of unlabeled chaperone from 1.5 to 6.25 µM were prepared. For studying homomeric CCH-CCH and CCHΔ-CCHΔ chaperone interaction with only one of the interaction partners pre-loaded with copper, copper loaded and labeled CCH was added to unlabeled CCH ranging from 0.4 to 6.25 µM. Accordingly, labeled CCHΔ was added to copper-loaded and unlabeled CCHΔ ranging from 0.4 to 6.25 µM. Equal volumes of the labeled chaperone and the corresponding dilution series were mixed resulting in the final concentrations given above. Samples were incubated for 10 min at room temperature in the dark prior to the measurement. Data were fit to a one-site binding model using the MO.Affinity Analysis software v2.1.5 (NanoTemper Technologies).

### Protein stability analysis by nanoDSF

Protein stability of CCH and CCHΔ was analyzed using the label-free nanoDSF method on a Prometheus NT.48 instrument (NanoTemper Technologies, Munich, Germany). All experiments were performed in triplicate at protein concentrations of 120 µM in SEC buffer. For experiments involving copper, samples additionally contained 30 µM CuCl, 75 µM BCA and 600 µM Na-ascorbate. Experiments analyzing the effect of the free copper ligand BCA on copper chaperone stability contained 75 µM BCA and 600 µM Na-ascorbate. In order to address the effect of the redox state on protein stability, melting temperatures (*T*_m_) were analyzed in the presence of 0.5 mM DTT and the absence of DTT. Samples did not contain DTT unless stated otherwise. Prior to the measurements, samples were transferred to nanoDSF-grade standard capillaries (NanoTemper Technologies, Munich, Germany). The start and end temperatures were set to 20 °C and 95 °C with an increase in temperature by 1.0 °C/min. The excitation power was set to 40% for samples with and without DTT and 70% for samples containing either the Cu(I)-(BCA)_2_ complex or the free BCA ligand due to lower signal intensities. The unfolding of proteins was monitored by calculating the fluorescence emission ratio at 330 nm and 350 nm and melting temperatures were determined from the inflection point of these ratios by the PR.ThermControl software (NanoTemper Technologies, Munich, Germany). Differences in melting temperatures were statistically analyzed by two-way ANOVA followed by pairwise multiple comparisons of means using the Bonferroni test.

### Isotope enrichment of CCH and NMR spectroscopy

Cells of *E. coli* strain BL21 (DE3) were transformed with the expression vector pETEV16b-CCH. Liquid 2YT media (100 ml) was inoculated with a single colony and grown overnight at 37 °C and 180 rpm. Cells were harvested by centrifugation at 7,500 × g for 15 min and the resulting pellet was resuspended in 30 ml M9 media supplemented with ^15^N-ammonium chloride and ^13^C-glucose. 6 × 500 ml M9 media supplemented with ^15^N-ammonium chloride and ^13^C-glucose were inoculated with 5 ml cell suspension and grown to an OD_600_ of 0.5 at 37 °C and 180 rpm. Cultures were cooled down to 16 °C and expression of CCH was induced by the addition of 0.5 mM Isopropyl β-D-1-thiogalactopyranoside (IPTG). Cells were harvested after 14 h of expression by centrifugation at 7500×*g* for 20 min and stored until purification at − 20 °C. Purification was carried out as described for unlabeled CCH^17^. NMR samples of apo-CCH contained 0.7 mM [U-^13^C, ^15^N]-CCH supplemented with 10 mM DTT added as a reducing agent, which would also chelate and deplete any residual copper that may have been carried over from the bacterial purification host, whereas Cu(I)-CCH samples contained 0.5 mM [U-^13^C, ^15^N]-CCH supplemented with an equimolar amount of Cu(I) as Cu(I)-(BCA)_2_ in 50 mM HEPES (pH 7.25) and 50 mM NaCl. For all experiments, 5 mm symmetrical Shigemi NMR tubes (BMS-005B) were used. NMR spectra were recorded at 25 °C on Bruker Avance III HD NMR spectrometers operating at 700 MHz and 750 MHz ^1^H Larmor frequencies equipped with cryogenically cooled TCI probes, as well as on a Bruker Avance III HD NMR spectrometer operating at 800 MHz equipped with a cryo-^13^C-TXO probe. The spectrometers were manually tuned and matched manually using the Bruker Tuning Module (ATMM) and shimmed using the TopShim routine. NMR samples contained [*U*-^13^C, ^15^N] or [*U*-^15^N] CCH in 50 mM HEPES (pH 7.25) and 50 mM NaCl supplemented with either 10 mM DTT or equimolar amounts of Cu(I) as Cu(I)-(BCA)_2_. Sequence-specific backbone assignments were obtained using band-selective excitation short-transient-transverse relaxation-optimized spectroscopy (BEST-TROSY) experiments^56^, Hadamard-encoded amino acid type editing (HADAMAC) ^57^ and ^13^C-detected (H)CANCO and (H)CANCOi experiments^58^. NMR data were processed using NMRPipe ^59^ and analyzed with CcpNmr^60^. The weighted chemical shift changes, Δδ(H,N), were calculated using the following Eq. (1):

1 $$\Delta \delta \left( {{}_{{}}^{1} H,{}_{{}}^{15} N} \right) = \sqrt {\Delta \delta \left( {{}_{{}}^{1} H} \right)^{2} + \frac{{\Delta \delta \left( {{}_{{}}^{15} N} \right)^{2} }}{25}}$$

Secondary chemical shifts were calculated on the basis of random-coil chemical shifts and corrected for next neighbor effects^61,62^.

### Structure prediction and molecular dynamics simulations of CCH dimers

To examine the evolutionary relationship of CCH from *A. thaliana* and its homologs in yeast and humans, the BLASTp (Basic Local Alignment Search Tool for proteins) server^40,41^ was employed. The full-length sequence of CCH from *A. thaliana* was obtained from the UniProt database (UniProt ID: O82089) and used as the query sequence. First, it was aligned with the amino acid sequence of ATX1 from *A. thaliana* (Uniprot ID: **Q94BT9**) using default parameters for homology assessment. Second, the query sequence was used to search against the PDB database^42^ using an expect threshold of 0.05, a word size of 3, a BLOSSUM80 matrix, and gap costs of 8 for existence and 2 for extension. The alignments were visualized using Jalview 2^63^, including all sequences with an *E*-value < 10^–4^. The CLUSTAL color scheme was used, and a diagram of sequence conservation and the consensus sequence were depicted. The homologous structures found were used to validate the *ColabFold* models created below.

No experimental 3D structure is currently available for CCH of *A. thaliana*. However, a structural model is available within the *AlphaFold* database^25^ (identifier: **O82089**)^64^. To generate a structural model of the corresponding CCH-dimer, *ColabFold* 1.5.2^39^ with default settings was used. Residues 72 to 121 of the CCH sequence were omitted to build CCH- and CCH∆-dimers since, based on the entire sequence, *ColabFold* failed to predict structures in which the cysteines 13 and 16 are facing towards each other as anticipated from homologous structures in the Protein Data Bank (PDB) ^42^ (*Saccharomyces cerevisiae*, Atx1-Cu(I)-Ccc2a complex PDB ID: **2GGP**)^45^, (*Homo sapiens*, COPPER-HAH1 PDB ID: **1FEE**)^27^ (Supplementary Figs. S3 and S4 online).

We further assessed the CCH- and CCH∆-dimers in the presence of the cofactor Cu(I) by unbiased molecular dynamics (MD) simulations. Since soluble copper chaperones such as CCH feature a similar structure consisting of a ferredoxin-like fold and a copper-binding motif (MxCxxC)^27,50^, the Cu(I) ion was placed between the sulfur atoms of cysteine residues Cys13 and Cys16 of the copper binding motif in the dimer, corresponding to the copper position in the homologs. The protein structures were further prepared and protonated using Maestro^65^ with pH 7.4 to mimic physiological conditions. The protonation states of the cysteines in the copper binding site were defined based on the homologs, too. According to PDB ID: **1FEE**, the dimer interaction is enhanced by an extended hydrogen bonding network, including two water molecules. When the hydrogen bonding network in PDB ID: **1FEE** is applied to CCH, one water molecule is located between the two sulfur atoms of cysteines Cys16_A_ and Cys16_B_. This water molecule also forms hydrogen bonds to Lys62_A_ and Lys62_B_. Therefore, Cys16_B_ is also assumed to be deprotonated. Additional hydrogen bonds connect Lys62_A_, Lys62_B_, a second water molecule, and the carbonyl oxygens of Thr60_A_ and Thr60_B_. Potential positions of water molecules in the dimerization interface were determined using 3D-RISM in AmberTools21^66,67^. The obtained structures were solvated in a rectangular box of transferable intermolecular potential with 3 points (TIP3P) water^68^ with a distance of at least 12 Å between the box edge and the protein. K^+^ ions were added as counterions in the solvation box. Counterions and copper were treated with the 12-6-4 non-bonded model^69^. The graphics processing units (GPU) particle mesh Ewald implementation from the AMBER22 molecular simulation suite^70–72^ with the ff14SB^73^ parameters for the protein were used.

The solvated systems were thermalized by carrying out a mixed steepest descent/conjugate gradient minimization with in total 50,000 steps, 50 ps of heating, and 50 ps of density adaptation with weak positional restraints on the protein’s atoms (2 kcal mol^−1^ Å^−2^) followed by an MD simulation under NPT conditions at 300 K for 4.9 ns. Covalent bonds to hydrogens were constrained using the SHAKE algorithm, and hydrogen mass repartitioning was applied which allows a time step of 4 fs^74,75^. The temperature was maintained by using Langevin dynamics^76^, with a friction coefficient of 2 ps^−1^. The pressure was maintained using an isotropic Berendsen barostat^77^. Production runs were started using the same setup as in the last step of thermalization. In total, 50 replicas were minimized and thermalized and then simulated for 200 ns each, resulting in a cumulative simulation time of 10,000 ns for both the CCH- and CCH∆-dimer. The generated trajectories were analyzed using CPPTRAJ^78^ or PYTRAJ of AmberTools^79^.

### MM-GBSA computations and free energy decomposition

To gain further insights into the CCH- and CCH∆-dimer interfaces, effective energy contributions of key residues that contribute significantly to the dimer's binding affinity were identified^55^. For this MM-GBSA^54^ calculations employing the “single trajectory approach” in MMPBSA.py of AMBER22^80^, considering gas-phase energies and solvation free energies but not configurational entropies^55^, were performed. The MM-GBSA calculations were based on 1000 snapshots extracted from the last 100 ns of equilibrated trajectories and performed using a modification on the “GBn” model (igb = 8) together with recommended radii set mbondi3. All counterions and water molecules were stripped, except the Cu(I) ion, whose radii were set to 1.4 Å^81^. To ensure an even distribution of the effective energy contributions of individual residues in both monomers, two cycles of calculations were performed, each time assigning the Cu(I) ion to a different monomer, with the results of both calculations being averaged. The contributions on a per-residue basis to the overall effective energy (i.e., sum of gas-phase plus solvation free energy) of dimerization are presented as Δ*G*_gas,solv_ averaged over the total of equilibrated independent trajectories. To display hot spots of the corresponding CCH-dimers, representative CCH-dimer structures were obtained by structural clustering.

### CCH-dimer and CCH∆-dimer versus monomer populations

The dimer-to-monomer equilibrium results from the following equilibrium:

$$2M \begin{array}{*{20}c} {K_{A} } \\ \rightleftharpoons \\ {K_{D} } \\ \end{array} \,\,\,D \ \ \ \ K_{D} = \frac{{\left[ M \right]^{2} }}{\left[ D \right]}$$

where D and M represent the CCH(∆)-dimer and monomer respectively, with *K*_D_ being the dimer dissociation constant, obtained from the experiment. Based on the measured dissociation constant of the CCH-dimer *K*_D_ = [M]^2^/[D] = 18 nM and the CCH∆-dimer *K*_D_ = [M]^2^/[D] = 57 nM, with [D] and [M] as concentrations of dimer and monomer, respectively, the proportion of CCH(∆)-dimer versus monomer in the cytosol of a cell can be calculated. To our knowledge, no in vivo CCH concentration has been measured so far; thus, we assume a concentration range of 10–1000 nM (see below and the "Results" section). An average Cu cell concentration of 6 mg L^−1^ is considered an appropriate concentration in *A. thaliana*^38^. This can increase to 20 mg L^−1^, but there is no experimental evidence that this also increases CCH expression^38^. This corresponds to a Cu concentration of 9.44 × 10^4^ nM:

$$n\left[ {Cu} \right] = \frac{mass \left( g \right)}{{molar \,mass \left( {\frac{{\text{g}}}{{{\text{mol}}}}} \right)}} = \frac{{6 \times 10^{ - 3} g}}{{63.55 \frac{{\text{g}}}{{{\text{mol}}}}}} = 9.44 \times 10^{ - 5} {\text{mol}}$$

$$c\left[ {Cu} \right] = 9.44 \times 10^{ - 5} \frac{{{\text{mol}}}}{{\text{L}}} = 94400 \frac{{{\text{nmol}}}}{{\text{L}}} = 9.44 \times 10^{4} \,{\text{nM}}$$

This is consistent with the Cu concentration of 7.0 × 10^4^ nM in *Saccharomyces cerevisiae*^14^. The concentration of the copper chaperone superoxide dismutase 1 (SOD1) in *S. cerevisiae* was measured as 10,000 nM^14^. As SOD1 is associated with mediating the copper transfer from the plasma membrane to the chloroplast^38^ and chloroplasts are one of the major Cu accumulation sites^38^, this suggests that fewer Cu ions need to be transported to the ER, and, accordingly, fewer copper chaperones (ATX1, CCH) mediating the copper transfer from the plasma membrane to the ER are needed. Interestingly, mRNA expression of CCH is increased in the absence of Cu and reduced with excess Cu, whereas ATX1 expression is induced by excess Cu^18^. This is in agreement with CCH mRNA expression measurements that suggest that CCH is present at a basal level in roots, stems, flowers, siliques, and leaves^19^, whereas SOD1 features high intracellular concentrations^14^. Based on this, and under consideration that both organisms feature a similar Cu cell concentration, we assume in the following that the intracellular concentration of CCH in *A. thaliana* is 10 to 1000 times lower than the concentration of SOD1 in *S. cerevisiae*. This results in a total concentration of CCH molecules of 10 to 1000 nM.

$$\left[ T \right] = 2\left[ D \right] + \left[ M \right] = \left[ {10, 1000} \right] \,{\text{nM}}$$

Expressing the dissociation constant in terms of the monomer concentration

$$K_{D} = \frac{{\left[ M \right]^{2} }}{{\frac{\left[ T \right] - \left[ M \right]}{2}}} \Leftrightarrow \left[ M \right]^{2} - \frac{{K_{D} \left[ T \right]}}{2} + \frac{{K_{D} \left[ M \right]}}{2} = 0$$

and solving the quadratic equation results in

$$\left[ M \right] = \frac{{\sqrt {K_{D} } \sqrt {K_{D} + 8\left[ T \right]} - K_{D} }}{4} = \left[ {6.0, 90.5} \right] \,{\text{nM }}\,\,\,for\,\,\, K_{D} = 18\,{\text{ nM}}$$

$$\left[ M \right] = \frac{{\sqrt {K_{D} } \sqrt {K_{D} + 8\left[ T \right]} - K_{D} }}{4} = \left[ {7.8, 155.2} \right] \,{\text{nM}}\,\,\, for\,\,\, K_{D} = 57\,{\text{nM}}$$

and

$$\left[ D \right] = \frac{\left[ T \right] - \left[ M \right]}{2} = \left[ {2.0, 454.8} \right]\,{\text{nM}}\,\,\,for\,\,\,K_{D} = 18\,{\text{nM}}$$

$$\left[ D \right] = \frac{\left[ T \right] - \left[ M \right]}{2} = \left[ {1.1, 422.4 } \right]\,{\text{nM}}\,\,\,for\,\,\,K_{D} = 57\,{\text{nM}}$$

The results show that in live cells the fraction of monomeric (dimeric) state is between 60 and 9% (40 and 91%) and 78 and 16% (22 and 84%) for full-length CCH (*K*_D_ = 18 nM) and CCH∆ (*K*_D_ = 57 nM), respectively.

### Supplementary Information

Supplementary Figures.

## Data Availability

NMR Data have been deposited at the BMRB under accession codes 50,358 and 50,359 or are available upon reasonable request to the corresponding author.

## Abbreviations

3D-RISM: 3D reference interaction site mode
ANOVA: Analysis of variance
ATOX1 (HAH1): Antioxidant 1 copper chaperone
ATX1: Antioxidant protein 1
BCA: Bicinchoninic acid
BCS: Bathocuproine disulfonate
BEST-TROSY: Band-selective excitation short-transient-transverse relaxation-optimized spectroscopy
CCH: Copper chaperone
CCP: Copper chaperone induced by pathogens
CD: Circular dichroism
CopZ: Copper chaperone Z from *Bacillus subtilis*
DTT: Dithiothreitol
*E. coli*: *Escherichia coli*
EPR: Electron paramagnetic resonance
ER: Endoplasmic reticulum
GPU: Graphics processing unit
HADAMAC: Hadamard-encoded amino acid type editing
HEPES: 2-[4-(2-Hydroxyethyl)piperazin-1-yl]ethane-1-sulfonic acid
His-tag: Polyhistidine-tag
HMA: Heavy metal-transporting ATPase
HSQC: Heteronuclear single quantum coherence
IMAC: Immobilized metal ion affinity chromatography
IPTG: Isopropyl β-D-1-thiogalactopyranoside
MD: Molecular dynamics
MDS: Microfluidic diffusional sizing
MM-GBSA: Molecular mechanics/generalized born surface area
MST: Microscale thermophoresis
n.s.: No significance
nanoDSF: Nano differential scanning fluorimetry
NHS-ester: *N*-Hydroxysuccinimide
NMR: Nuclear magnetic resonance
PAE: Predicted alignment error
PDB: Protein data bank
pLDDT: Predicted local distance difference test
RAN1: Responsive to antagnoist 1
RMSD: Root-mean-square deviation
ROS: Reactive oxygen species
s.d.: Standard deviation
SEC: Size exclusion chromatography
SOD: Superoxide dismutase
SPR: Surface plasmon resonance
TEV: Tobacco etch virus
TIP3P: Transferable intermolecular potential with 3 points
Tris/HCl: Tris(hydroxymethyl)aminomethane hydrochloride
UV: Ultraviolet
