# Supplementary material for: Structure and dimerization properties of the plant-specific copper chaperone CCH
Source: Sci Rep. 2024 Aug 17;14:19099. doi: 10.1038/s41598-024-69532-y (PMC11330527; doi:10.1038/s41598-024-69532-y)
Supplement: Supplementary file 1 — Supplementary Figures. [file 41598_2024_69532_MOESM1_ESM.pdf]

# Supplementary Information

# Structure and dimerization properties of the plant-specific copper chaperone CCH

Dominik Dluhosch[1], Lisa Sophie Kersten[2], Stephan Schott-Verdugo[3], Claudia Hoppen[1], Melanie Schwarten[4], Dieter Willbold[4,5], Holger Gohlke[2,3] and Georg Groth[1*]

[1]Institute of Biochemical Plant Physiology, Heinrich-Heine-Universität Düsseldorf, 40225 Düsseldorf, Germany

[2]Institute for Pharmaceutical and Medicinal Chemistry, Heinrich-Heine-Universität 40225 Düsseldorf, Düsseldorf, Germany

[3]Institute of Bio- and Geosciences: Bioinformatics (IBG-4), Forschungszentrum Jülich, 52425 Jülich, Germany

[4]Institute of Biological Information Processing: Structural Biochemistry (IBI-7), Forschungszentrum Jülich, 52425 Jülich, Germany

[5]Institut für Physikalische Biologie, Heinrich-Heine-Universität Düsseldorf, 40225 Düsseldorf, Germany

*Corresponding author:
Georg Groth, Biochemical Plant Physiology, Heinrich-Heine-Universität Düsseldorf, Universitätsstraße 1, 40225 Düsseldorf, Tel. 0049 211 81-12822,
E-mail: georg.groth@hhu.de

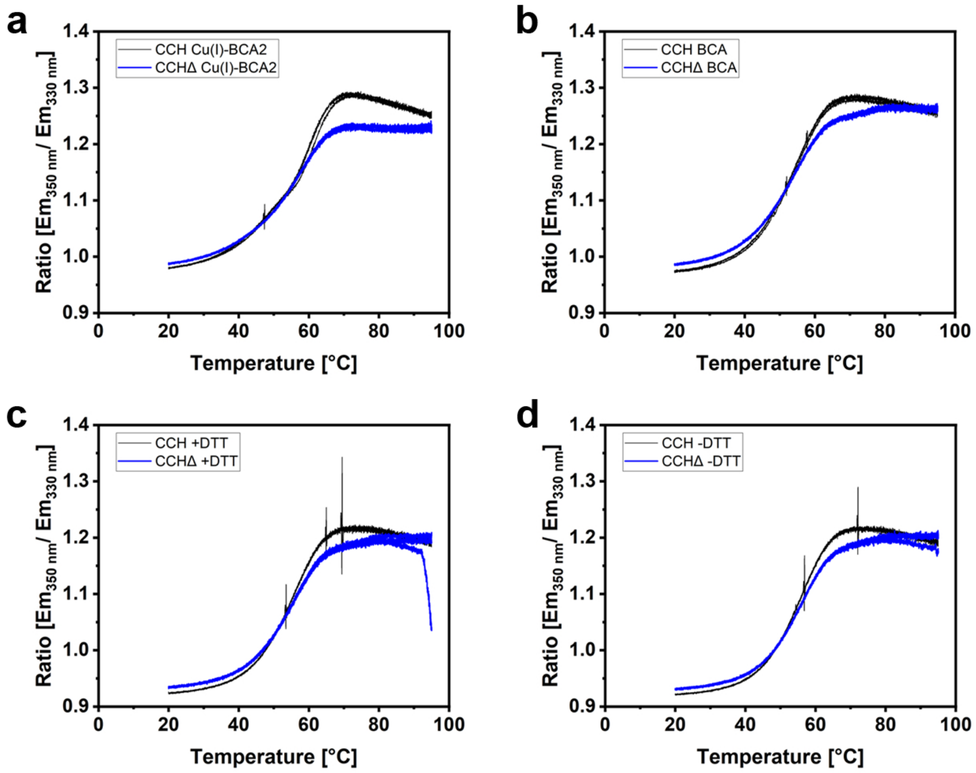


**Supplementary Fig. S1. Melting curves of CCH and CCHΔ determined by nanoDSF.** The fluorescence emission of CCH and CCHΔ at 350 nm and 330 nm is plotted as the 350 nm/330 nm emission ratio as a function of temperature to monitor temperature dependent protein unfolding. Spectra of CCH (black) and of CCHΔ (blue) in the presence of (a) Cu(I)-$(BCA)_2$, (b) copper-free BCA, (c) DTT and (d) without DTT. In each figure, spectra of all three technical replicates are shown.

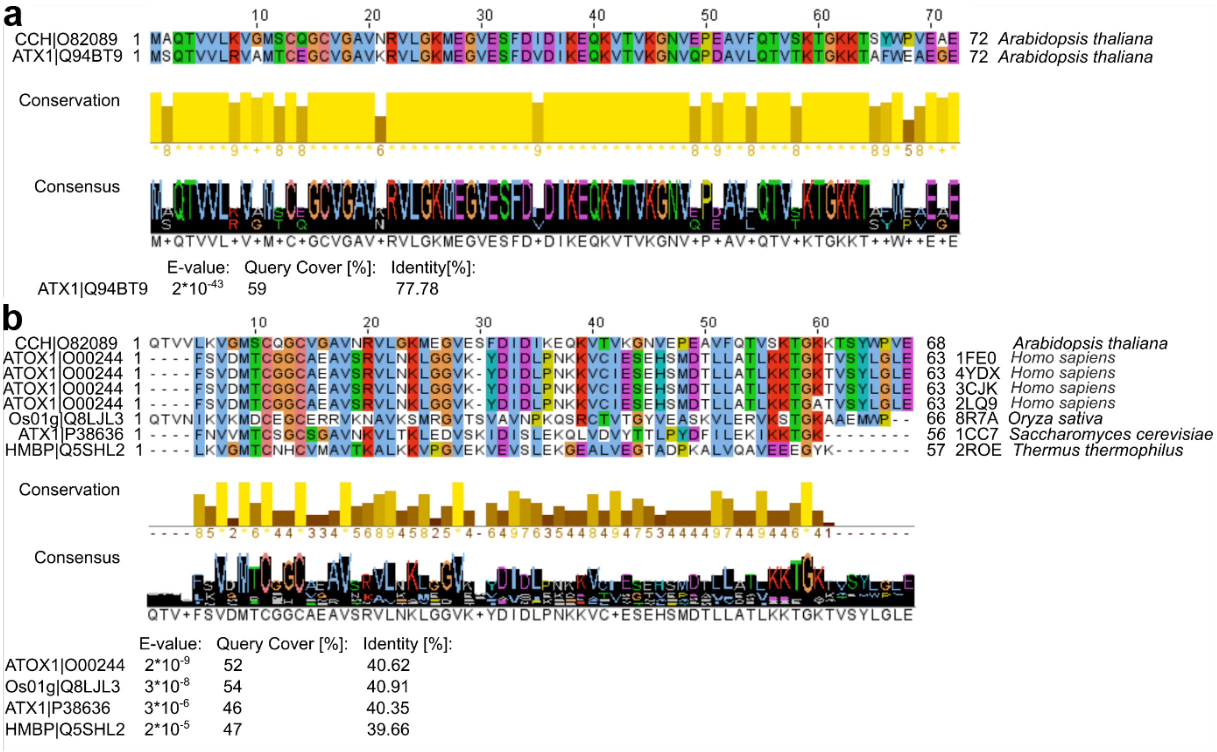


**Supplementary Fig. S2. Sequence alignment of CCH to known homologs.** Amino acids are colored according to the CLUSTAL color scheme. (a) Alignment of CCH and ATX1 in *A. thaliana*. The alignment has an E-value of $2x10^{-43}$, a query coverage of 59 %, and a sequence identity of 78 %. (b) Alignment of CCH to sequences of homologous structures from the PDB database. The E-value, query coverage, and sequence identity range from $2x10^{-9}$ to $3x10^{-6}$, 54 % to 46 %, and 41 % to 39 %, respectively. CCH shares several conserved amino acids, including the characteristic copper-binding motif (MxCxxC) with the homologous structures, confirming CCH as a homolog of ATX1 from *A. thaliana*, yeast, and humans.

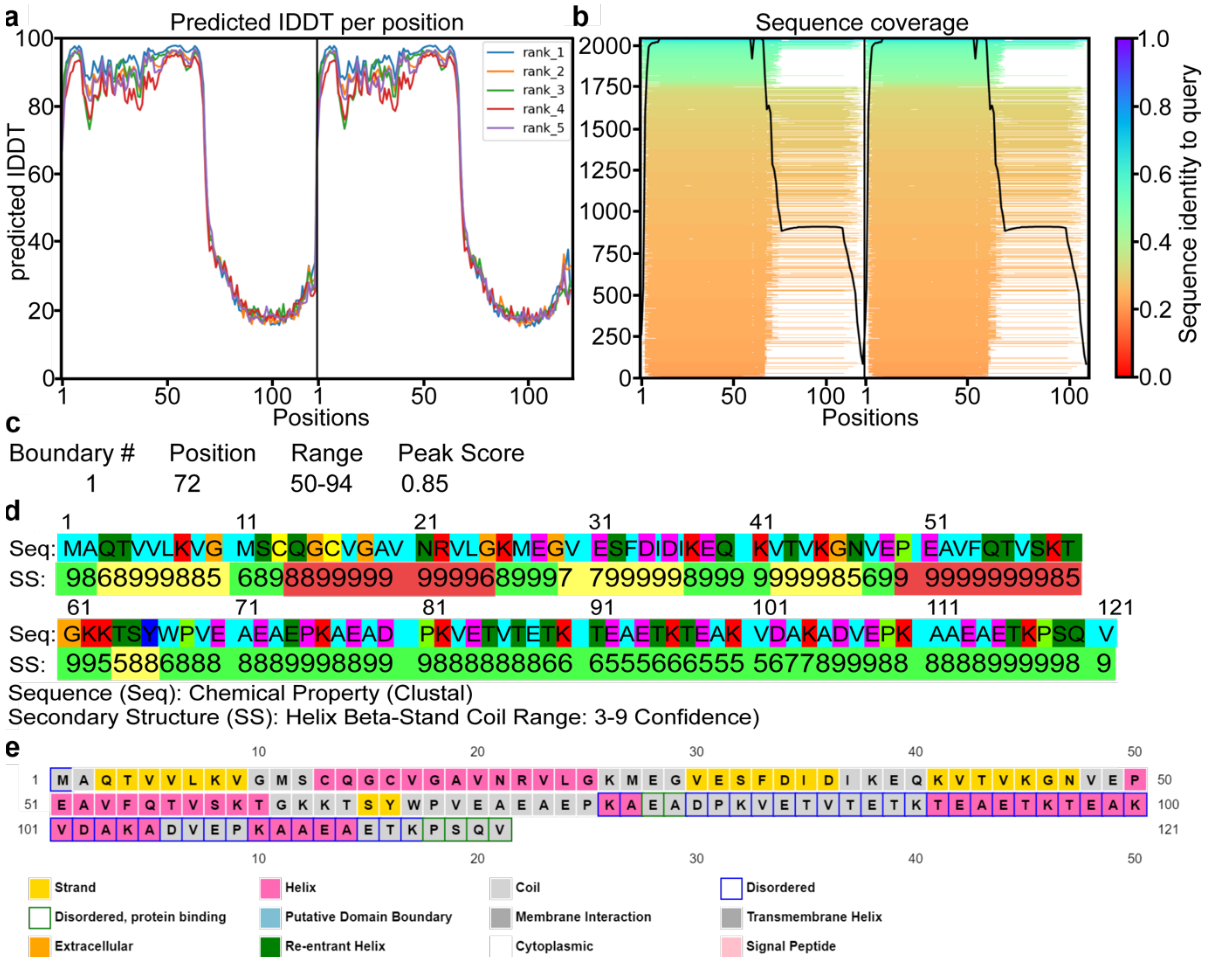


**Supplementary Fig. S3. Analysis of CCH dimer prediction.** (a) pLDDT (per-residue Local Distance Difference Test) of the CCH-dimer predicted by *ColabFold*. The pLDDT value provides an estimate of a residue's local accuracy of the predicted protein structure on a scale of 0 to 100. A value of 70 to 90 indicates a good model, as is the case for the ATX1-like fold, where there is a generally good prediction of the backbone. Since a pLDDT value below 50 is present for the C-terminal end, this prediction should not be interpreted further. (b) Sequence coverage of the CCH-dimer predicted by *ColabFold*. High sequence coverage indicates that a significant portion of the protein structure has been predicted, leading to a more comprehensive understanding of the three-dimensional arrangement of the protein; this is the case for the ATX1-like fold of CCH. The lower sequence coverage in the C-terminal part of CCH indicates that the structure prediction is less reliable to unavailable. (c) TopDomain prediction of a putative domain boundary at position 72 of the full-length CCH sequence with a peak of 0.8. (d) TopProperty prediction of the CCH structure. Residues 1-66 form the ATX1-like fold. The C-terminus (residues 67-121) is predicted to be an unstructured coil. The sequence is colored according to CLUSTAL colors for chemical properties; the secondary structure (SS) is in red for helices, yellow for β-strands, and green for coil conformation. (e) DISOPRED prediction of the CCH structure. Similar to TopProperty, residues 1-66 form the ATX1-like fold. The C-terminus is predicted to be disordered.

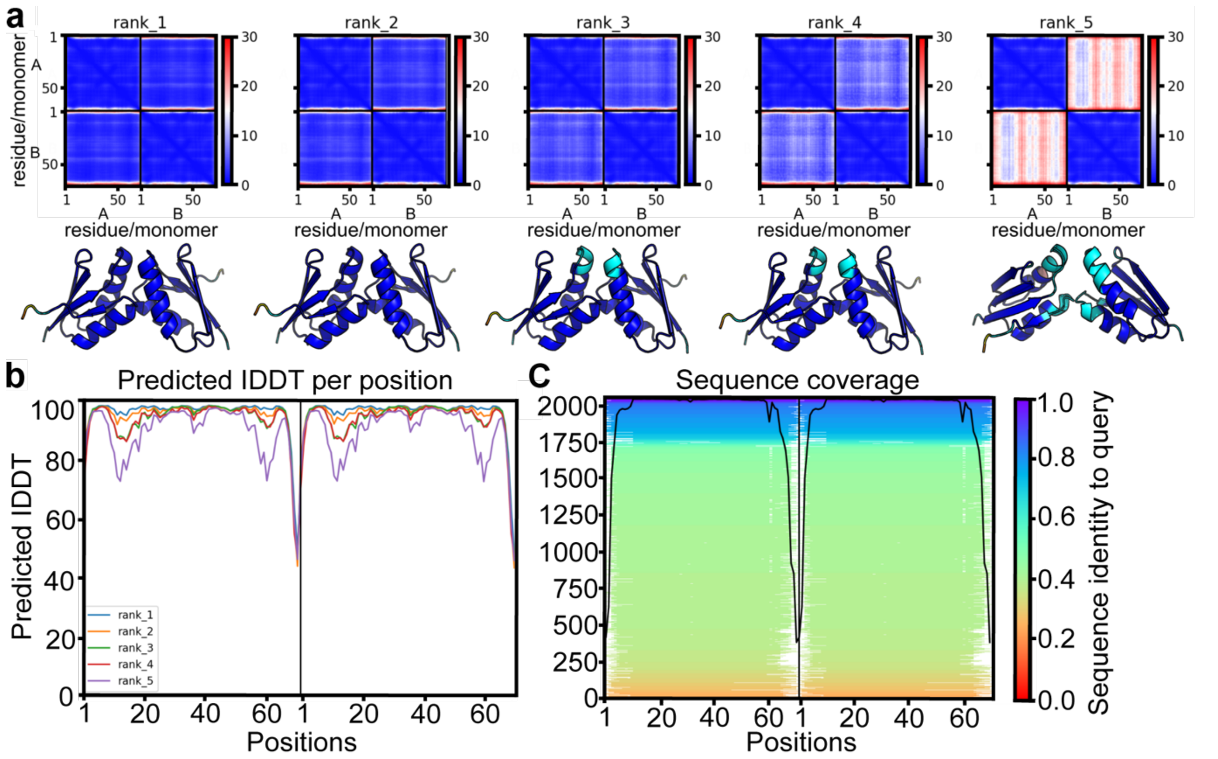


**Supplementary Fig. S4. Quality analysis of the CCHΔ dimer model.** (a) Predicted alignment error (PAE) matrices of the CCHΔ-dimer models from *ColabFold*. A high PAE (red) indicates a high expected position error for the residue at x, if the predicted and true structure is aligned at residue y. A low PAE (blue) indicates low expected position errors. The vertical and horizontal lines in the PAE matrix separate the corresponding monomers forming the CCHΔ-dimer. Structures of the corresponding ranks are colored according to pLDDT. Regions with pLDDT > 90 are expected to be modeled with high accuracy (dark blue). Regions with pLDDT of 70 to 90 are expected to be modeled well (turquoise). A pLDDT between 50 and 70 indicates low confidence (orange). (b) Display of pLDDT per position and (c) sequence coverage for the CCHΔ-dimer. Residues 1-66 show a low PAE and a high model confidence, indicating an overall good quality of the CCHΔ-dimer.

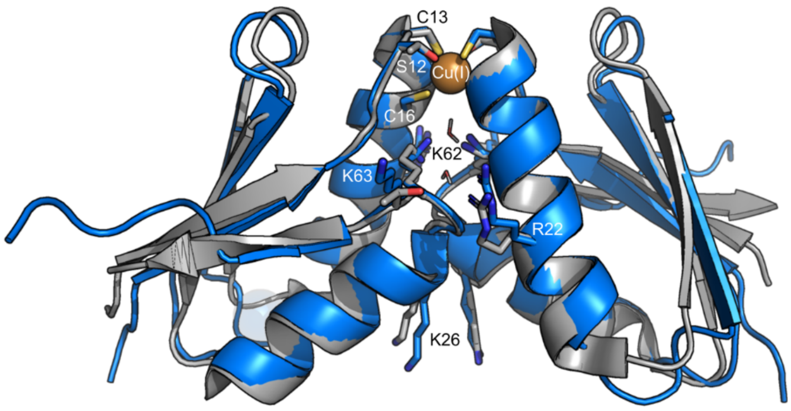


**Supplementary Fig. S5. Comparison of the CCHΔ-dimer and a crystal structure of the CCH homologue HAH1 dimer bound to Cu(I).** Overlay of the *ColabFold* CCHΔ-dimer model (blue) and the X-ray structure of the CCH homolog HAH1 (PDB ID: **1FEE**, grey). Although both proteins share a sequence identity of 41%, the Cα RMSD between the X-ray structure and the CCHΔ-dimer is 0.5 Å. Amino acids forming the copper binding motif and the dimer interface are mostly conserved in both proteins. Furthermore, the orientation of the side chains known to form the dimer interface is broadly consistent between the model and PDB ID: **1FEE**. This is particularly true for the residues near the copper binding site S12, C13, and C16. Minor deviations exist for residues R22, K26, K62, and K63.

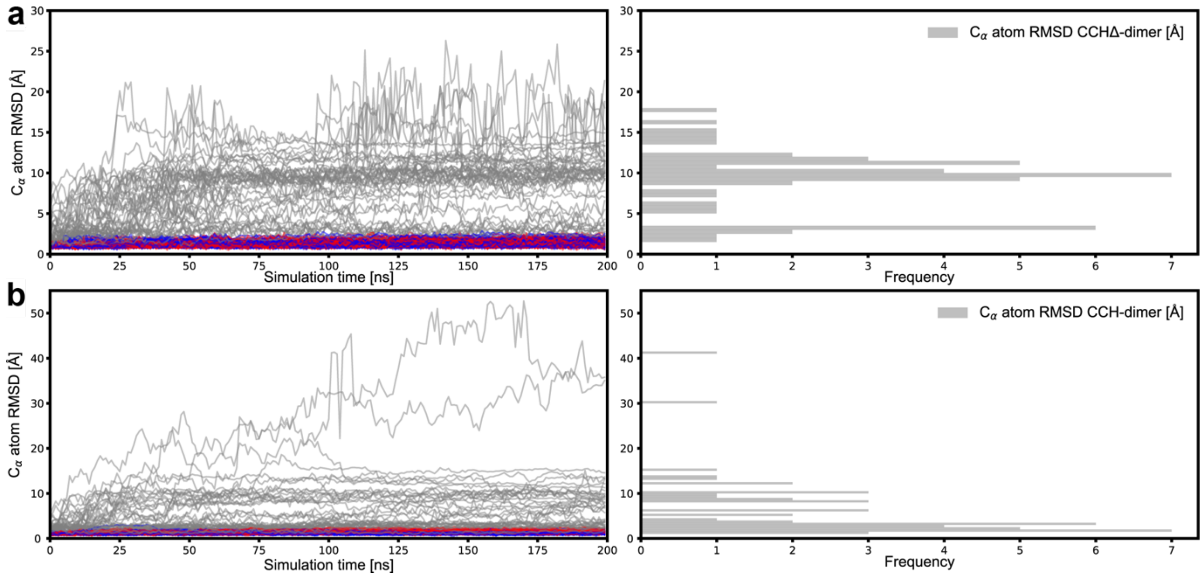


**Supplementary Fig. S6. Cα atom RMSD of each ATX1-like fold and the dimer overall.** (a) Left: Cα atom RMSD of each ATX1-like fold (red and blue) and the CCHΔ-dimer (grey) of all 50 replicas. Right: Distribution of the corresponding mean Cα atom RMSD values from nanosecond 101 to 200 of the CCHΔ-dimer. (b) Left: Cα atom RMSD of each ATX1-like fold (red and blue) and the CCH-dimer (grey) of all 50 replicas. Right: Distribution of the corresponding mean Cα atom RMSD values from nanosecond 101 to 200 of the CCH-dimer.

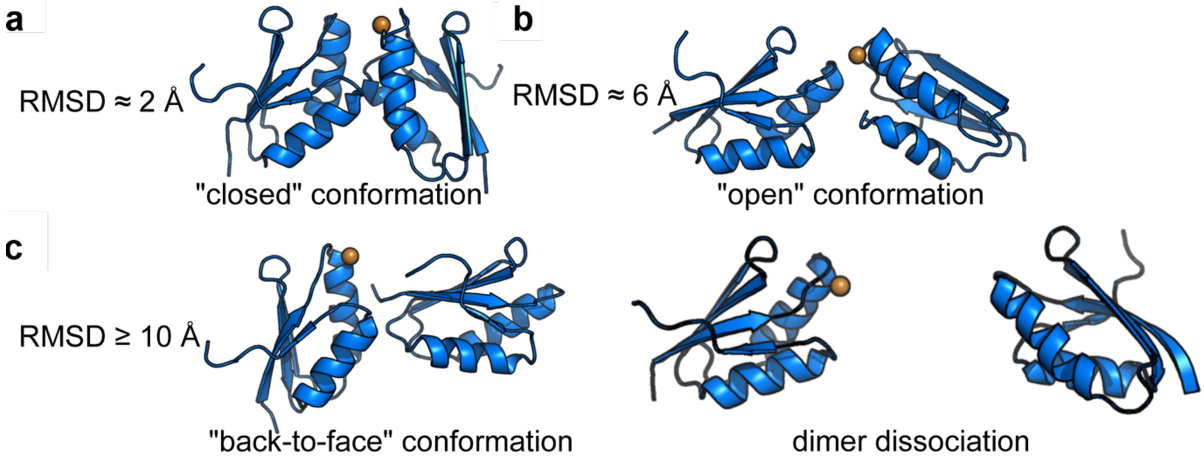


**Supplementary Fig. S7. Different states of CCH-dimers that occur in CCH- and CCHΔ-dimer trajectories.** (a) Representative of a CCH-dimer structure for replicas showing a Cα RMSD of approximately 2 Å. This structure is comparable to the “closed” conformation of the homolog ATOX1. (b) Representative of a CCH-dimer structure of replicas showing a Cα RMSD of approximately 6 Å. This structure is comparable to the “open” conformation of the homolog ATOX1. (c) Representation of CCH-dimer structures of replicas showing a Cα RMSD of ≥ 10 Å. The structures are similar to the “back-to-face” conformation of the homolog ATOX1 or show dimer dissociation.

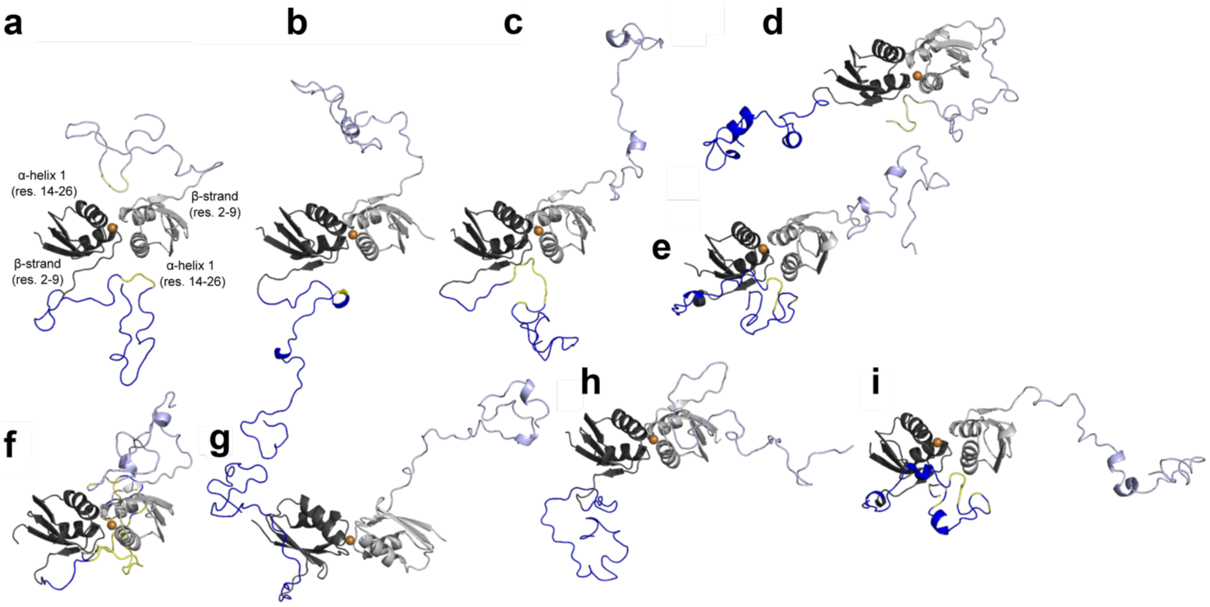


**Supplementary Fig. S8. Molecular dynamics simulations of CCH with emphasize on the structural arrangement of the C-terminal extension.** Representative structures of spatial arrangements of the C-termini (dark and light blue) in copper-loaded CCH-dimers (ATX1-like folds in dark and light grey). No interaction of the C-terminal ends with each other was observed. Either the C-termini are exposed to the solvent (panels b, g, h) or located close to the monomer at the same side as the first β-strand (see labels) of the ATX1-like fold. Yellow colored residues show less than 5 Å distance to the first α-helix (residues 14-26) of the other monomer (panels a, c, d, e, f, i).

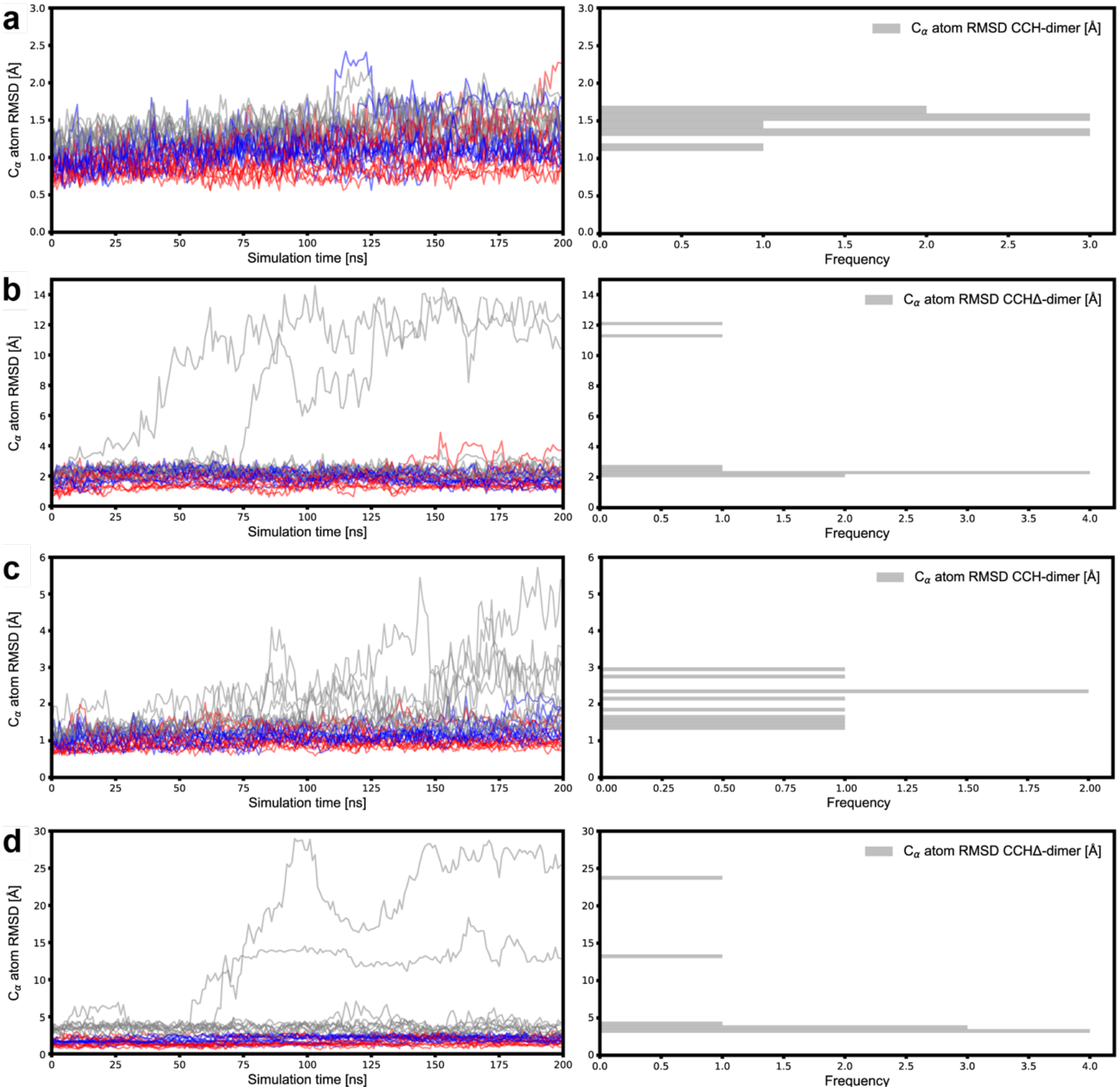


**Supplementary Fig. S9. Cα atom RMSD of each ATX1-like fold and the dimer overall after restart.** (a) Restarting the final frame in replica 43 results in 10 out of 10 replicas with an overall RMSD < 4 Å. Left: Cα atom RMSD of each ATX1-like fold (red and blue) and the CCH-dimer (grey) of all 10 replicas. Right: Distribution of the corresponding mean Cα atom RMSD values from nanosecond 101 to 200 of the CCH-dimer. (b) Restarting the final frame in replica 49 results in 8 out of 10 replicas with an overall RMSD < 4 Å. Left: Cα atom RMSD of each ATX1-like fold (red and blue) and the CCHΔ-dimer (grey) of all 10 replicas. Right: Distribution of the corresponding mean Cα atom RMSD values from nanosecond 101 to 200 of the CCHΔ-dimer. (c) Restarting the final frame in replica 43 results in 6 out of 10 replicas with an overall RMSD < 4 Å. Left: Cα atom RMSD of each ATX1-like fold (red and blue) and the CCHΔ-dimer (grey) of all 10 replicas. Right: Distribution of the corresponding mean Cα atom RMSD values from nanosecond 101 to 200 of the CCH-dimer. (d) Restarting the final frame in replica 49 results in 8 out of 10 replicas with an overall RMSD < 4 Å. Left: Cα atom RMSD of each ATX1-like fold (red and blue) and the CCHΔ-dimer (grey) of all 50 replicas. Right: Distribution of the corresponding mean Cα atom RMSD values from nanosecond 101 to 200 of the CCHΔ-dimer.
